# Pre-clinical validation of a pan-cancer CAR-T cell immunotherapy targeting nfP2X7

**DOI:** 10.1038/s41467-023-41338-y

**Published:** 2023-09-08

**Authors:** Veronika Bandara, Jade Foeng, Batjargal Gundsambuu, Todd S. Norton, Silvana Napoli, Dylan J. McPeake, Timona S. Tyllis, Elaheh Rohani-Rad, Caitlin Abbott, Stuart J. Mills, Lih Y. Tan, Emma J. Thompson, Vasiliki M. Willet, Victoria J. Nikitaras, Jieren Zheng, Iain Comerford, Adam Johnson, Justin Coombs, Martin K. Oehler, Carmela Ricciardelli, Allison J. Cowin, Claudine S. Bonder, Michael Jensen, Timothy J. Sadlon, Shaun R. McColl, Simon C. Barry

**Affiliations:** 1 Molecular Immunology, Robinson Research Institute, University of Adelaide, Adelaide, SA 5000 Australia; 2 Chemokine Biology Laboratory, Department of Molecular and Cellular Biology, School of Biological Sciences, University of Adelaide, Adelaide, SA 5005 Australia; 3 University of South Australia, STEM (Future Industries Institute) SA, Adelaide, 5095 Australia; 4 Centre for Cancer Biology, University of South Australia and SA Pathology, Adelaide, SA 5001 Australia; 5 Reproductive Cancer Research Group, Discipline Obstetrics and Gynaecology, Robinson Research Institute, University of Adelaide, Adelaide, SA 5005 Australia; 6 Seattle Children’s Research Institute, Seattle, WA 98101 USA; 7 Carina Biotech, Level 2 Innovation & Collaboration Centre, UniSA Bradley Building, Adelaide, SA 5001 Australia; 8 Department of Gynaecological Oncology, Royal Adelaide Hospital, Adelaide, SA 5005 Australia; 9 Adelaide Medical School, The University of Adelaide, Adelaide, SA 5005 Australia; 10 Department of Gastroenterology, Women’s and Children’s Health Network, North Adelaide, SA 5006 Australia

**Keywords:** Immunology, Cancer immunotherapy

## Abstract

Chimeric antigen receptor (CAR)-T cell immunotherapy is a novel treatment that genetically modifies the patients’ own T cells to target and kill malignant cells. However, identification of tumour-specific antigens expressed on multiple solid cancer types, remains a major challenge. P2X purinoceptor 7 (P2X7) is a cell surface expressed ATP gated cation channel, and a dysfunctional version of P2X7, named nfP2X7, has been identified on cancer cells from multiple tissues, while being undetectable on healthy cells. We present a prototype -human CAR-T construct targeting nfP2X7 showing potential antigen-specific cytotoxicity against twelve solid cancer types (breast, prostate, lung, colorectal, brain and skin). In xenograft mouse models of breast and prostate cancer, CAR-T cells targeting nfP2X7 exhibit robust anti-tumour efficacy. These data indicate that nfP2X7 is a suitable immunotherapy target because of its broad expression on human tumours. CAR-T cells targeting nfP2X7 have potential as a wide-spectrum cancer immunotherapy for solid tumours in humans.

## Abstract (web summary)

The scarcity of targetable proteins broadly expressed on cancer cells, but not on healthy cells, is an obstacle for chimeric antigen receptor (CAR)-T cell therapy. Here the authors establish that a functionally impaired version of P2X purinoceptor 7, non-functional P2X7 (nfP2X7), fulfils these criteria, and demonstrate that CAR-T cells targeting nfP2X7 efficiently and selectively kill breast and prostate cancer cells in mouse models.

## Introduction

Immunotherapy—enabling a cancer patients’ own immune cells to recognize and attack their tumours, represents a paradigm shift in the treatment of cancer. This shift is towards a personalised treatment that does not require broad therapies such as chemotherapy to achieve clinical endpoints. These immunotherapies arm immune cells to specifically recognise and destroy malignant cells, which requires tight regulation to prevent collateral damage to healthy tissues. One immunotherapy approach showing much promise is chimeric antigen receptor (CAR)-T cell therapy, which combines the antigen-specific binding properties of monoclonal antibodies with the killing and self-renewal capacity of T cells. The elegant simplicity of the CAR-T cell platform is that it combines the ability to target unprocessed antigens using antibody-like binding domains in an MHC-independent manner with the intracellular signalling required to initiate cytotoxic activity in T cells. Recent clinical trials of CD19-targeting CAR-T cell therapy have demonstrated impressive results in treating a variety of haematological malignancies, with complete remission rates of up to 80–93% in patients with relapsed or refractory B-cell acute lymphoblastic leukaemia (B-ALL)^1,2^, and long-term remission of 20 months in adult patients with a low disease burden of relapsed B-ALL^3^. CD19-targeting CAR-T cell therapy has also achieved significant clinical responses in patients with chronic lymphocytic leukaemia (CLL) and non-Hodgkin’s lymphoma^4–7^.

Robust CAR-T cell clinical activity against these haematological cancers has inspired the search for tumour-specific antigens, which will enable the generation of CAR-T cells capable of attacking a broad range of other cancers, especially solid tumours. However, clinical trials with CAR-T cell therapies against solid tumours have yielded disappointing results, as demonstrated by a meta-analysis of CAR-T cell therapy against solid tumours that revealed a 9% overall response rate across 22 clinical trials^8^. The limited response of CAR-T cell therapies towards solid tumours in clinical trials may be due to a number of factors, including the quality of the CAR-T cells, inefficient homing of CAR-T cells and immunosuppression within the tumour microenvironment. Tumour heterogeneity is frequently observed in solid cancers and is another issue that may hinder CAR-T cell therapy, causing tumour escape and patient relapse^9^. In addition, the selection of target antigens which are only found on the malignant cells is challenging. While administration of CD19-targeting CAR-T cell therapy results in the elimination of both healthy and malignant B cells, causing B cell hypoplasia, this on-target off-cancer cytotoxicity can be clinically managed. Of the limited selection of antigens expressed by solid cancers investigated to date, many are either also present on healthy cells in a variety of tissues, risking on-target off-cancer cytotoxicity and organ damage, or are present in only a small number of cancers. To avoid serious adverse effects due to on-target off-tumour cytotoxicity or restricted application, it is critical to find and validate CAR-T targets that are absent on healthy cells, and preferably are expressed by a wide range of solid tumours.

P2X purinoceptor 7 (P2X7) is an ATP-gated cation channel which is widely expressed on many tissues and on haemopoietic cells^10,11^. When functioning normally, P2X7 controls ion transport in response to ATP, in which short-term ATP binding results in the opening of the cation-selective channel to allow Na^+^ and Ca^2+^ influx and K^+^ efflux. This drives downstream signalling pathways involved in cytokine release, cell survival and proliferation. Under prolonged binding to ATP in ATP-rich conditions, P2X7 functions as a non-selective pore and becomes permeable to large molecules (<900 Da), which results in programmed cell death^12,13^. This results in a dichotomous response in that activity of the cation channel can promote either survival or death of the cell.

Several studies have characterised a non-functional version of P2X7, named nfP2X7. Critically, the adoption of the nfP2X7 conformation exposes a unique epitope in the extracellular domain that is normally buried in the internal structure of the protein. Antibodies that recognise this epitope have been developed, and these have revealed significant overexpression of nfP2X7 on many solid tumours, including those originating from the breast, ovary, brain, prostate, skin, bowel, ovary, cervix, lung, pancreas, and stomach. In contrast, the expression of nfP2X7 is undetectable on healthy cells, including healthy cells within the proximal tissue of nfP2X7-positive tumour specimens^12,14–16^. NfP2X7 is thought to represent a conformationally constrained non-functional form of the receptor, which retains its ion transporter function without pore enlargement^12,14^. As a result, cell death through this receptor is blocked, conferring a selective advantage on transformed cells. This may allow nfP2X7-positive cells to avoid apoptotic cell death in ATP-rich conditions such the tumour microenvironment, where ATP released by dying cells accumulates to extracellular concentrations in the hundreds of micromolar range^14^. The expression of nfP2X7 on a diverse range of tumour cells is consistent with the notion that the receptor conveys a selective advantage on cancer cells, which is an attractive attribute for a CAR-T target^12,14^. Indeed, siRNA-mediated knockdown of nfP2X7 on the nfP2X7-positive prostate cancer cell line, PC3 resulted in apoptotic cell death^12^.

To evaluate the potential of nfP2X7 as a broad range solid tumour target, we constructed a second-generation CAR against nfP2X7 using an affinity matured peptide binding domain that binds to an exposed epitope on nfP2X7. Here, we demonstrate that nfP2X7-targeting CAR-T cells generated from multiple donors display broad anti-tumour efficacy against twenty-four cancer cell lines in vitro, representing twelve different cancer types. We confirm on target specificity by selectively deleting the *P2X7* gene, and nfP2X7-targeting CAR-T cells show reduced cytotoxicity against the deleted cells. We demonstrate that nfP2X7-targeting CAR-T cells exhibit robust in vivo anti-tumour efficacy and long-term survival in triple-negative human breast cancer and prostate cancer xenograft mouse models, and nfP2X7-targeting CAR T cells can traffic to, and infiltrate the tumour parenchyma, preferentially accumulating within tumours. The significance is that expression of nfP2X7 on a diverse range of cancers is a major advantage of targeting nfP2X7 to generate a potentially universal CAR-T cell therapy. These results position nfP2X7-targeting CAR-T cells as a potential broad-spectrum immunotherapy against solid tumours in humans.

## Results

### Linker length impacts the antigen-specific cytotoxicity of nfP2X7-CAR-T cells

**Fig. 1:**
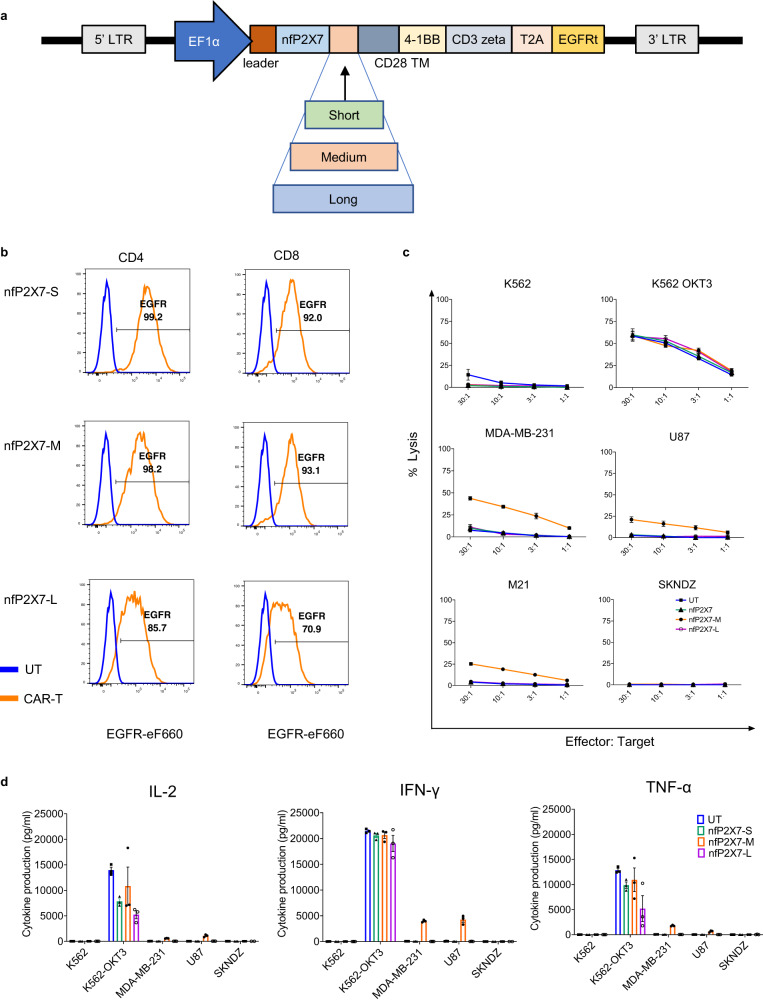
Impact of the hinge length on cytotoxicity potential of nfP2X7-CAR-T cells. **a** Schematic of the lentiviral vector construct with EF1α promoter followed by the leader sequence, nfP2X7 antigen binding domain, hinge region (short, medium or long), CD28 transmembrane domain, 41BB, CD3 zeta, T2A and EGFRt. **b** Flow cytometric analysis of CAR expression in CD4^+^/CD8^+^ compartments on the three different CAR-T cells (nfP2X7-S, nfP2X7-M and nfP2X7-L) by EGFR staining. **c** Specific cytotoxicity of target cells by CD8^+^ nfP2X7-CAR-T cells with three different hinge lengths (nfP2X7-S, nfP2X7-M and nfP2X7-L) compared with CD8^+^ untransduced (UT) cells. Target cell lines include: K562 (leukaemia), MDA-MB-231 (breast cancer), U87 (glioma), M21 (melanoma) and SK-ND-Z (neuroblastoma). K562 transduced to express OKT3 was included as a positive control. CAR-T cells were co-cultured with target cancer cell lines at effector: target ratios of 30:1, 10:1, 3:1 and 1:1 for 4 h. Specific cytotoxicity was measured using a chromium-51 release cytotoxicity assay. Pooled data from two independent experiments. Data are presented as mean values +/− SEM. **d** Cytokine release assay for nfP2X7-CAR-T cells. CD4^+^ CAR-T cells and target cells were co-cultured for 24 h and the concentration of IL-2, IFN-ɣ and TNF-α in the supernatant was measured using the Bio-Plex system. Cytokines produced by untransduced CD4^+^ cells were compared with nfP2X7-CAR-T cells harbouring different hinge lengths. Target cell lines include: K562, K562-OKT3, MDA-MB-231, U87 and SK-ND-Z. Data represent two independent experiments. Data are presented as mean values +/− SEM.

To generate a nfP2X7-targeting CAR, a previously described and validated nfP2X7 peptide-binding domain^12,15–17^ was cloned into a second-generation CAR lentiviral backbone encoding a IgG4 hinge/linker and intracellular domains from 41BB and CD3 zeta, connected by a T2A self-cleaving peptide to a truncated EGFR (EGFRt) reporter^2,18–20^. As the target epitope must be captured within a functional immunological synapse, the tertiary structure of the extracellular domain of the CAR impacts the antigen binding and activity of the CAR-T cell. The hinge region is used to connect the antigen-binding domain to the transmembrane domain, and the hinge length can play an important role in optimal CAR activity. Therefore, to optimize the tertiary structure of the nfP2X7-targeting CAR, we tested three different hinge lengths to find the most effective combination for maximum cytotoxicity (Fig. 1a). The following coding was used to identify each CAR-T construct: nfP2X7-S (IgG4 hinge), nfP2X7-M (IgG4 hinge-CH3) and nfP2X7-L (IgG4 hinge-CH2-CH3). The long hinge used here comprised a modified CH2 domain to prevent binding to FCɤ receptors which can cause off-target cytotoxicity^21^. To determine the optimum linker length, donor-matched CD8^+^ and CD4^+^ cells were isolated as separate populations and transduced with lentiviruses encoding the nfP2X7-specific CAR with one of three different hinge lengths. Following T cell expansion, transduction efficiency was assessed by EGFR reporter expression. All constructs were transduced into T cells at a high efficiency, with a larger percentage of nfP2X7-S (99.2% of CD4^+^, 92.0% of CD8^+^) and nfP2X7-M (98.2% of CD4^+^, 93.1% of CD8^+^) cells expressing EGFR. The nfP2X7-L construct displayed a comparatively lower expression of EGFR (85.7% of CD4+, 70.9% of CD8^+^) (Fig. 1b).

As it has been well-established that CD8^+^ T cells mediate direct tumour cytolysis while CD4^+^ T helper cells provide cytokines to support CD8^+^ T cell cytotoxicity, the cytotoxic potential of CD8^+^ T cells was assessed in in vitro cytotoxicity assays, and cytokine production of CD4^+^ T cells was assessed in cytokine release assays. The cytotoxicity potential of CD8^+^ CAR-T cells, which varied according to hinge length, was compared using a ^51^Cr release assay against five cancer cell lines: MDA-MB-231 (breast cancer), U87 (glioma), M21 (melanoma), SK-ND-Z (neuroblastoma) and K562 (leukaemia). As a positive control, we used K562 cells that were transduced to express the anti-CD3 antibody, OKT3. Briefly, CAR-T cells were co-cultured with target cancer cell lines at effector cell: target cell (E:T) ratios of 10:1, 3:1 and 1:1 for 4 h. Untransduced donor-matched CD8^+^ T cells prepared in parallel alongside CAR-T cells from the same donor, were used as a negative control to measure non-specific cytotoxicity. All CD8^+^ cells demonstrated high levels of cytolytic activity against K562-OKT3 cells (positive control), indicating that all (CAR-T and untransduced) populations were able to mount an effective cytolytic responses as a result of polyclonal activation. Out of the three CAR-T cell populations tested, nfP2X7-M CAR-T cells showed significant dose-dependent cytotoxic activity towards MDA-MB-231 (43%), U87 (25%), and M21 (21%) cell lines at a E:T ratio of 30:1 but did not demonstrate activity against SK-ND-Z or K562 cells (Fig. 1c). The other two CAR-T cell formulations (nfP2X7-S or nfP2X7-L) did not show in vitro antigen-specific cytotoxicity against any of the tumour cell lines tested despite showing comparable levels of transgene expression and cytotoxic response to the control target cells (K562-OKT3). Together, these data indicate that hinge length significantly impacts the antigen-specific cytotoxicity of nfP2X7-targeting CAR-T cells in vitro.

Furthermore, the production of T_H_1 cytokines by CD4^+^ CAR-T cells, which harboured the three different hinge lengths, was assessed. All CD4^+^ CAR-T cell populations demonstrated equivalent cytokine production when co-cultured with the positive control K562-OKT3 cell line. Significant levels of IL-2, IFN-ɣ and TNF-α were detected in supernatants of CD4^+^ nfP2X7-M-CAR-T cells co-cultured with MDA-MB-231 and U87 cancer cell lines, but not when co-cultured with either SK-ND-Z or K562 cells (Fig. 1d). This was consistent with the cytotoxic function of CD8^+^ nfP2X7-M CAR-T cells observed in vitro. Furthermore, minimal concentrations of T_H_1 cytokines were detected in the culture media from CD4^+^ T cells expressing nfP2X7-S or nfP2X7-L in response to co-culture with cancer cells, which was also consistent with the results of the cytotoxicity assays with CD8^+^ CAR-T cells. Based on the enhanced antigen-specific cytotoxicity and cytokine release profile of the nfP2X7-M CAR, we selected this CAR for all subsequent in vitro and in vivo experiments.

### nfP2X7-CAR-T specific killing of a broad range of cancer cell lines in vitro

**Fig. 2:**
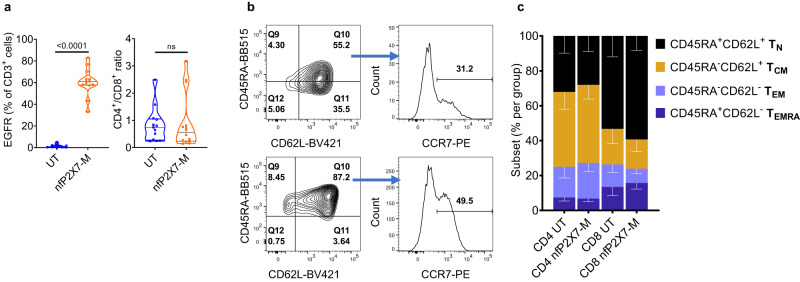
NfP2X7-CAR-T cells consist of mostly naive and central memory phenotypes. **a** Frequency of EGFR reporter expression on total CD3^+^ cells 7 days post-transduction (*n* = 13), ratio of CD8^+^/CD4^+^ of CD3^+^ end of expansion on d14 post-PBMC; *n* = 13. Data is pooled from 13 independent experiments each dot represents an experiment conducted with T cells from a separate donor. **b** Representative FACS plots for CD45RA^+^, CD62L^+^, and CCR7^+^ expression of CD4^+^ and CD8^+^ nfP2X7-M CAR-T cells at end of expansion on d14 post-PBMC. **c** Frequency of T_SCM_, T_Naive_, T_CM_, T_EM_ and T_EMRA_ and populations based on CD45RA^+^, CD62L^+^ and CCR7^+^ expression; *n* = 7. Pooled data from seven independent experiments. Data represents mean ± SEM. Statistical significance was evaluated by paired T test. ns: non-significant.

To further investigate the cytotoxicity potential of the nfP2X7-M CAR-T cells, we tested these cells against a more comprehensive range of cancer cell lines, including 24 different cancer cell lines from 12 cancer types. From this point onwards, bulk CD3^+^ T cells were used for CAR-T cell production. This strategy was selected as it yields transduced CD4^+^ and CD8^+^ in a single transduction protocol. Using this combined CD3^+^ T cell protocol, consistently high transduction efficiency (on average 60%) by the CAR lentivirus was observed (Fig. 2a). The CD8^+^/CD4^+^ ratio on d14 post-stimulation with PBMC skewed towards a higher frequency of CD8^+^ T cells, with a 0.82:1 mean CD8^+^:CD4^+^ ratio in the untransduced populations and a 0.9:1 mean CD8^+^:CD4^+^ ratio in the CAR-T cell populations (Fig. 2a). As the normal CD8^+^/CD4^+^ ratio in human peripheral blood is 1:1.75 to 1:2.3 (Supplementary Fig. 6a)^22^, these data indicate that the skewing toward CD8^+^ T cells is a consequence of the manufacturing process. A selection of surface and intracellular markers was used to define cytotoxic potential, the stage of cell maturation, activation, and co-inhibitory molecule expression for all preparations of untransduced control and CAR-T cells (Supplementary Table 2, Supplementary Figs. 3–5). After expansion, T cells comprised of a heterogeneous population consisting of mostly naive-like (T_N_, CD45RA^+^ and CD62L^+^), central memory (T_CM_, CD45RA^-^ and CD62L^+^) and stem cell memory (T_SCM_, CD45RA^+^, CD62L^+^, CCR7^+^ and CD95^+^) subset phenotypes. All cells were CD95+ (Supplementary Fig. 7a) There were also some effector memory (T_EM_, CD45RA^-^ CD62L^-^) and effector memory RA (T_EMRA_, CD45RA^+^ CD62L^-^) T cells present in both the untransduced control and CAR-T cell populations (Fig. 2b, c). The short duration of CAR-T cell culture expansion (14 days) retained a large percentage of naive-like and central memory phenotypes (Fig. 2b, c). Significant expression of the activation markers (CD27, CD28, CD95 and CXCR3) was detected in CAR-T cell populations at the end of expansion (Supplementary Fig. 7a). Furthermore, minimal expression of the co-inhibitory markers CTLA-4, PD-1 and LAG-3 (Supplementary Fig. 7b) and high expression of the Th1 cytokines IFN-γ, IL-2, TNF-α and cytotoxicity marker granzyme B was observed (Supplementary Fig. 8a–c). Importantly, comprehensive characterisation of untransduced control T cells and CAR-T cells showed no significant differences between the populations. To demonstrate antigen-specific binding by the CAR construct, we stained untransduced and CAR-T cells with a biotin-labelled peptide mimetic of the nfP2X7 epitope. To correlate the frequency of CAR-T cells that were EGFR^+^ and were capable of binding to the nfP2X7, simultaneous surface staining with anti-EGFR and the nfP2X7 peptide mimetic was performed. The frequency of stained cells using either reagent were not significantly different, demonstrating EGFR expression is a reliable reporter of CAR expression (Supplementary Fig. 9a, b). These data also indicated that nfP2X7-targeting CD3^+^ CAR-T cells were capable of specifically recognising the cognate target antigen and displaying highly antigen-specific killing.

Taken together, nfP2X7-targeting CAR-T cells were transduced at a high transduction efficiency and displayed a favourable T cell phenotype, characterized by minimal differentiation and co-inhibitory molecule expression and high frequencies of activation and cytotoxic molecule expression.

**Fig. 3:**
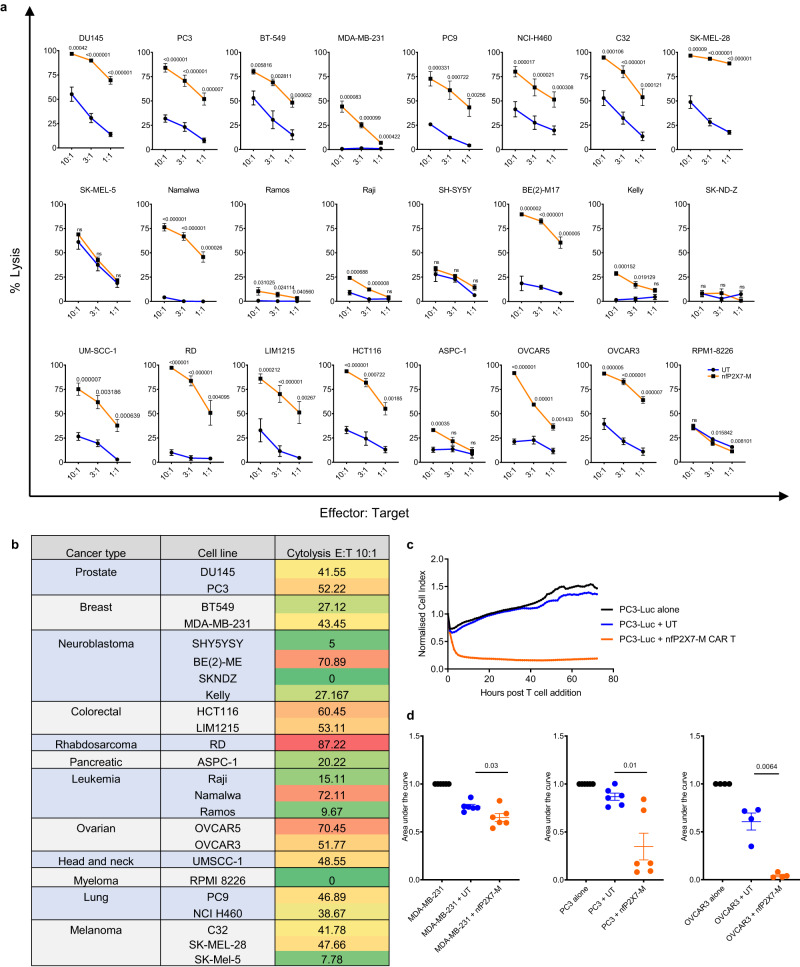
NfP2X7-CAR-T cells exhibit broad-spectrum cytotoxicity against a diverse range of cancer cells. **a** Cytotoxicity of target cells by CD3^+^ nfP2X7-M-CAR-T cells compared with donor-matched untransduced (UT) cells generated in parallel. Target cell lines include: DU145 (prostate cancer), PC3 (prostate cancer), BT-549 (breast cancer), MDA-MB-231 (breast cancer), PC9 (lung cancer), NC1-H460 (lung cancer), C32 (melanoma), Sk-Mel-28 (melanoma), Sk-Mel-5 (melanoma), Namalwa (lymphoma), Ramos (lymphoma), Raji (lymphoma), SH-SY5Y (neuroblastoma), Be(2)-M17 (neuroblastoma), Kelly (neuroblastoma), SK-ND-Z (neuroblastoma), UM-SCC-1 (head and neck carcinoma), RD (rhabdomyosarcoma), LIM1215 (colorectal cancer), HCT116 (colorectal cancer), AsPC-1 (pancreatic cancer), OVCAR5 (ovarian cancer), OVCAR3 (ovarian cancer), RPMI-8226 (myeloma). CAR-T cells were co-cultured with target cancer cell lines at effector:target E:T ratios of 10:1, 3:1 and 1:1 for 16 h. Specific cytotoxicity was measured using a BrightGlo luciferase-based cytotoxicity assay system; paired T test comparing the nfP2X7-M CAR-T with CD3^+^ UT at the indicated time points. Pooled data from three independent experiments. Statistical significance was evaluated by paired T test. Exact *p* values on the graph, ns: non-significant. **b** Table listing cancer types and cancer cell lines tested. Cytotoxicity values at E:T ratio of 10:1, with cytotoxicity values from untransduced control T cells subtracted. **c** Representative graph showing changes in normalised cell index over time (in hours) for the human prostate cancer cell line PC3 when untreated (black) or treated with either untransduced T cells (UT) (dark blue) or nfP2X7-M CAR T cells (orange). Black vertical line reflects time point at which T cells were added. **d** The area under the curves were measured and normalised to the cancer cell alone control. Data represents mean ± SEM. Data is pooled from independent experiments for MDA-MB-231 (*n* = 6), PC3 (*n* = 6) and OVCAR3 (*n* = 4) target cell lines, where each dot represents an experiment conducted with T cells from a separate donor. Statistical significance was evaluated by paired T test. Exact *p* values on the graph, ns: non-significant.

**Fig. 4:**
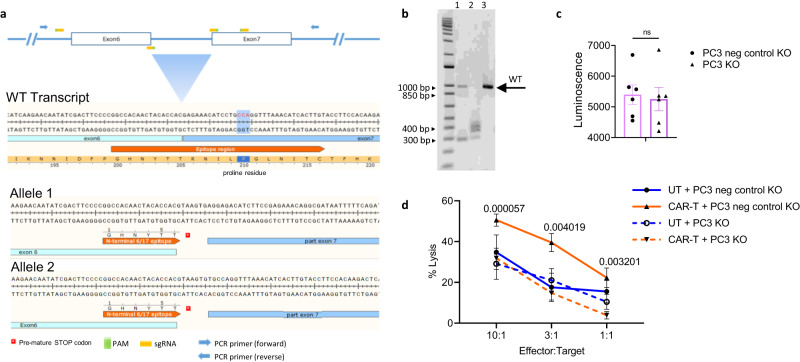
NfP2X7-CAR-T cell-mediated cytotoxicity is dependent on P2X7 expression by tumour cells. **a** A schematic representation of the CRISPR Cas9 targeting strategy to generate a PC3 prostate cancer *P2X7* Knock out (KO) clone with the position of the four sgRNA molecules used for targeted homologous recombination denoted by orange boxes and the PAM sequence denoted by green boxes. Flanking PCR primers are indicated by blue arrows. Specific targeting of both alleles of the human P2X7 locus in exon 6 was verified by sequencing. In the WT transcript row (row 1), the target epitope sequence GHNYTTRNILPGLNITC is highlighted and the proline residue in the 17 amino acid epitope required for nfP2X7-M CAR-T cell binding is annotated. Rows 2 and 3 show that the N-terminal proportion of the epitope is retained followed by a frameshift and premature stop coding (red asterisk) in alleles 1 and 2, respectively. **b** Representative agarose gel of PCR products generated from single-cell cloning of knockout pools. Heterozygous clone (lane 1), knockout clone 21 (lane 2) and WT (lane 3, single band) are shown. Individual PCR products were gel purified and cloned into the pCR4-TOPO TA vector and sequenced. The expected WT fragment is marked by an arrow. **c** Total bioluminescence signal from viable cells in the luciferase cytotoxicity assay for PC3 KO negative (neg) control and PC3 *P2X7* KO cells, transduced to express luciferase and incubated with luciferin. PC3 KO negative control cells were transduced with a non-targeting sgRNA sequence. Data are presented as mean values +/− SEM. **d** Cytotoxicity assays testing lysis of PC3 *P2X7* KO and PC3 KO negative control cells. CAR-T or donor-matched untransduced T cells (UT) were co-cultured with target cancer cells at effector:target ratios of 10:1, 3:1 and 1:1 for 16 h. Specific cytotoxicity was measured using a BrightGlo luciferase-based cytotoxicity assay system. Statistical significance was evaluated by paired T test comparing nfP2X7-M CAR-T-mediated cytotoxicity against PC3 *P2X7* KO cells with PC3 KO negative control cells at the indicated effector to target ratios. Exact *p* values on the graph, ns: non-significant. Data pooled from three independent batches of CAR-T cells generated from three donors. Data are presented as mean values +/− SEM.

To determine the cytotoxicity potential of the nfP2X7-M CAR-T on multiple cancer types we next used a luminescence-based cytotoxicity assay. Briefly, effector CAR-T cells were co-cultured with cancer cells (target cells) stably expressing luciferase at E:T ratios of 10:1, 3:1 and 1:1 for 16 h. Significantly higher CAR-T cell-specific cytotoxicity was observed compared with untransduced control cells against the majority of the cancer cell lines tested. The highest level of cytotoxicity was observed against the C32 (melanoma), SK-MEL-28 (melanoma), Be(2)-M17 (neuroblastoma) and RD (rhabdomyosarcoma) cell lines. A titration-dependent effect was also observed, with percentage cytotoxicity values decreasing as the E:T ratio decreased (Fig. 3a). Some cancer targets were more efficiently killed by the CD3^+^ nfP2X7-M CAR-T cells than others, which is summarised as a heatmap of tumour cell lysis percentage at the E:T of 10:1 (Fig. 3b). Taken together, these data show that nfP2X7-targeting CAR-T cells are cytotoxic against 22 out of 24 different cancer cell lines tested in vitro, demonstrating broad cancer specificity. Furthermore, real-time cytotoxicity assays were performed using the xCELLigence impedance system to assess the kinetics of CAR-T cell cytotoxicity in vitro. Co-culture of nfP2X7-M CAR T cells with three human cancer cell lines (MDA-MB-231, PC3, OVCAR3) resulted in significantly lower impedance of tumour cells, represented as a normalized cell index, compared with untransduced donor-matched control T cells. This demonstrated that CAR-T cells mediate significant and prolonged anti-tumour cytotoxicity against cancer cell lines, which resulted in almost complete elimination of cancer cells after 70 h co-culture (Fig. 3c, d). To further confirm that nfP2X7-M CAR-T cells act via the *P2X7* gene locus and possess on-target specificity, we targeted the nfP2X7 epitope (GHNYTTRNILPGLNITC) using CRISPR gene editing. To this end, we generated a PC3 clone in which a premature stop codon was introduced into both alleles (GHNYTT*) (Fig. 4a, b), and generated a PC3 cell line transduced with a non-targeting sgRNA as the negative control (referred to as PC3 KO negative control). We confirmed that the PC3 KO negative control and PC3 *P2X7* KO cells had equivalent viability via bioluminescence (Fig. 4c). In in vitro cytotoxicity assays, we observed a significant 40–60% reduction in nfP2X7-M CAR-T cell mediated-cytotoxicity to the PC3 *P2X7* KO cells compared to PC3 KO negative control cells. These results were consistent between three independent batches of nfP2X7-M CAR-T cells generated from three donors. We also assayed the cytotoxicity driven by donor-matched untransduced cells, and the background cytotoxicity against both PC3 KO negative control and PC3 *P2X7* KO cells was not significantly different from the nfP2X7-M CAR-T cells co-cultured with PC3 *P2X7* KO cells, suggesting that nfP2X7-M CAR-T-mediated cytotoxicity was dependent on expression of P2X7 (Fig. 4d).

To investigate potential off-target activity of nfP2X7-targeting CAR-T cells, in vitro cytotoxicity assays were performed using normal healthy PBMCs. PBMCs were selected as wild-type P2X7 was reported to be expressed on many haematopoietic cells including monocytes, macrophages, B cells, T cells, dendritic cells and natural killer cells^11,23^. To test in vitro cytotoxicity using the luciferase-based method, PBMCs were first transduced with the lentivirus, LV-GFP-Luc to stably express firefly luciferase, and cytotoxicity mediated by nfP2X7-M-CAR-T cells or untransduced control CD3^+^ T cells was assessed as previously described. In this assay, even at the highest dose of nfP2X7-M CAR-T cells (effector:target, 10:1), no cytotoxicity against PBMC cells was detectable (Supplementary Fig. 10a). Mined gene expression data from The Human Protein Atlas confirms many different cell types of PBMCs express P2X7, as shown by sequencing of both single cells and flow-sorted populations of PBMCs (Supplementary Fig. 10b, c). These data indicate that nfP2X7-targeting CAR-T cells do not respond to the functional wild-type form of P2X7.

### nfP2X7-targeting CAR-T cells engage with and elicit effector function against cancer cells in vitro and in vivo

**Fig. 5:**
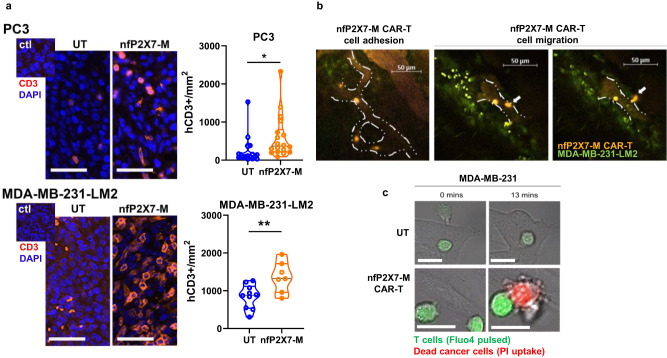
CAR-T cells are readily detected within tumours of NSG mice. **a** 2 × 10^6^ MDA-MB-231-LM2 or PC3 cells were injected into NSG mice (mammary fat pad or flank, respectively) and allowed to establish for 2 weeks prior to the mice receiving an intravenous injection of 2 × 10^7^ untransduced (UT) or nfP2X7-M CAR-T cells. Tumours were harvested at 12–14 days post T cell injection, FFPE, and 3–5 sections from throughout the tumour were stained with an anti-human CD3^+^ antibody and DAPI. Representative images shown with the secondary alone control included as inserts. PC3 UT *n* = 3 sections/tumour from 6 tumours (2 tumours per mouse). MDA UT *n* = 5 sections/tumour from 2 mice, CAR-T *n* = 4 sections/tumour from 2 tumours (1 tumour per mouse). Scale bars = 50 µm. The number of CD3^+^ DAPI^+^ cells were quantified, with groups blinded. Graph shows the mean ± SEM CD3^+^ DAPI^+^ T cells in MDA-MB-231-LM2 tumours (*n* = 4–5 sections from each of the *n* = 2 tumours/mice from each group) or PC3 tumours (*n* = 3 sections from each of the *n* = 6 tumours harvested from 3 mice/group, with two tumours per mouse) receiving UT or nfP2X7-M CAR-T cells; *p < 0.05, **p < 0.01, unpaired T-test. **b** For intravital microscopy, 2 × 10^6^ MDA-MB-231-LM2 cells expressing GFP were injected into the mammary fat pad of NSG mice and allowed to establish for 2 weeks. The mice then received an intravenous injection of 2 × 10^7^ CAR T nfP2X7-M cells (labelled with CellTracker Orange) as well as Cy5 Dextran (for visualisation of blood vessels with active blood flow). Zeiss LSM710 with a 20x objective lens was used to visualise the surgically exposed tumour. Representative images showing CAR-T cells firmly adhered to a blood vessel (left panel) and a CAR-T cell transmigrating across a blood vessel wall (middle and right panels) (*n* = 2). Scale bars = 50 µm. **c** T cell killing was also observed using in vitro real-time imaging techniques. MDA-MB-231-LM2 cells were seeded into wells and allowed to grow overnight. T cells pulsed with Fluo4 AM (green) and PI (red) to detect dying cells were then added into the wells and images were taken at 10 s intervals for at least 3 h using a 40x objective lens on the Zeiss LSM700. Representative images shown from two independent experiments.

To determine whether nfP2X7-targeting CAR-T cells infiltrate a solid tumour in vivo, we injected MDA-MB-231-LM2 breast cancer cells into the mammary fat pad of NSG mice or PC3 prostate cancer cells into the flank of NSG mice and allowed tumours to form for 12 days prior to i.v. injection of untransduced or nfP2X7-M CAR-T cells. Tumours were harvested approximately 2 weeks post-T cell injection and processed for immunofluorescence analysis to determine human CD3^+^ (hCD3^+^) T cell numbers. There were significantly more hCD3^+^ DAPI^+^ T cells within the PC3 and MDA-MB-231-LM2 tumours of mice treated with nfP2X7-M CAR T cells when compared to the mice treated with untransduced T cells (Fig. 5a). These results indicate that, while both nfP2X7-M CAR T cells and untransduced T cells were able to traffic to and infiltrate the tumour parenchyma, nfP2X7-M CAR T cells preferentially accumulated within tumours, which may be attributed to increased retention, survival and/or proliferation. To gain further insight into the possible interactions between CAR-T cells and tumours in vivo, intravital microscopy was performed on anaesthetised mice harbouring orthotopic GFP-tagged MDA-MB-231-LM2 tumours. Tumours were allowed to establish for approximately 2 weeks prior to i.v. injection of 50 kDa Cy5 Dextran to visualise the tumour vasculature. At this point, nfP2X7-M CAR-T cells labelled with CellTracker Orange were administered into the mice and tumours were immediately imaged by multi-photon microscopy. As shown in representative still images (Fig. 5b), nfP2X7-targeting CAR-T cells were identified as circulating cells in the tumour vasculature (white outline) within 2 min post-administration. Moreover, nfP2X7 CAR-T cells were observed to adhere to the vascular walls (Fig. 5b, left panel, Supplementary Movie 1) and transmigrate though the endothelial cell wall (Fig. 5b, centre and right panels, Supplementary Movie 2). Furthermore, in vitro real-time imaging revealed that nfP2X7-M CAR-T cells were able to directly engage with and promote tumour cell death (Fig. 5c, Supplementary Movie 3), while untransduced cells exhibited almost no tumour cell engagement and cytotoxicity (Fig. 5c, Supplementary Movie 4).

### nfP2X7-targeting CAR-T cells inhibit human tumour xenograft growth in vivo

**Fig. 6:**
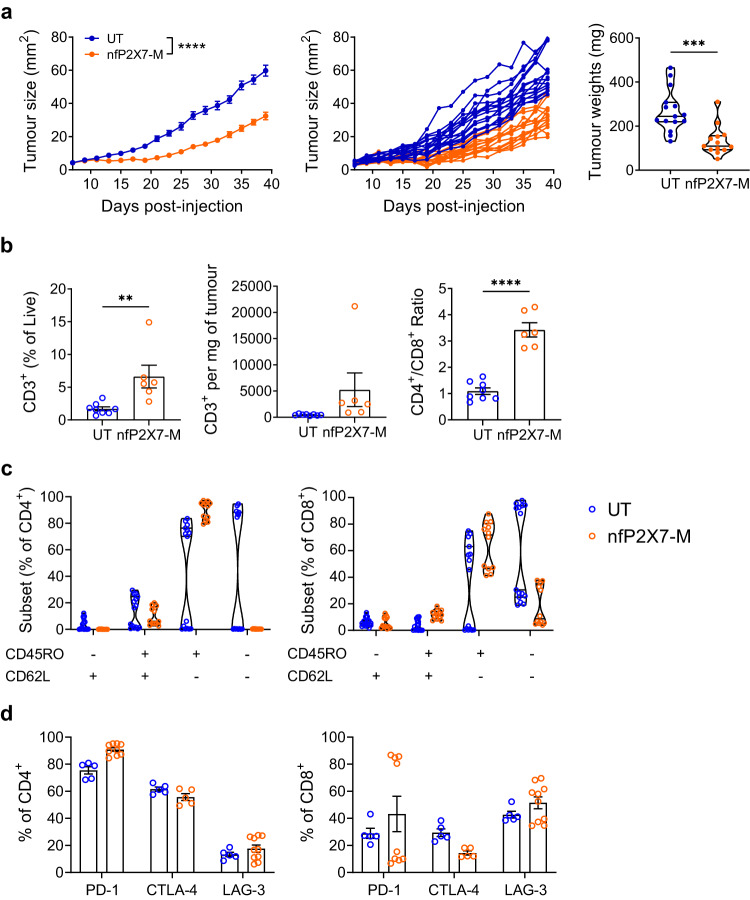
NfP2X7-targeting CAR-T cells significantly inhibit the tumourigenesis of a human breast cancer xenograft model. 6–8-week-old female NOD-*scid* IL2Rγ^null^ (NSG) mice were subcutaneously injected with 2 × 10^6^ MDA-MB-231 human breast cancer cells into the fourth mammary fat pad and intravenously injected with 2 × 10^7^ nfP2X7-targeting CAR-T cells or untransduced T cells on d3 post-tumour injection. Tumours were harvested for flow cytometric analysis at d40 post-tumour injection. **a** Tumour growth curves (as pooled and individual mice) and endpoint tumour weights; *n* = 15 (UT) and *n* = 13 (nfP2X7-M). Tumour size: two-way ANOVA with Bonferroni’s post-test, ****p < 0.0001; tumour weight: two-tailed unpaired *t*-test, ***p = 0.0003. **b** Frequency of human CD3^+^ T cells of total viable cells in tumours, number of human CD3^+^ T cells per mg of tumour and CD4^+^/CD8^+^ ratio of CD3^+^; *n* = 8 (UT) and *n* = 6 (nfP2X7-M); two-tailed unpaired *t-*test, **p = 0.0071, ****p < 0.0001. **c** Frequencies of T cell subsets as defined by CD45RO and CD62L expression by intratumoural CD4^+^ and CD8^+^ T cells; *n* = 15 (UT) and *n* = 12 (nfP2X7-M); two-way ANOVA with Bonferroni’s post-test. **d** Frequency of PD-1, CTLA-4 and LAG-3 expression by intratumoural CD4^+^ and CD8^+^ T cells; *n* = 5 (UT PD^-^1, CTLA-4, LAG-3) and *n* = 9 (nfP2X7-M PD-1), *n* = 5 (nfP2X7-M CTLA-4), *n* = 10 (nfP2X7-M LAG-3). Data in (**a**, **c**, **d**) are pooled from 2 independent experiments following in vivo delivery of 2 independent CAR-T cell preparations derived from 1 healthy donor and data in (**b**) representative of a single independent experiment. Data represented as mean ± SEM.

To investigate the in vivo anti-tumour efficacy of nfP2X7-M CAR-T cell therapy, CAR-T cells were delivered into two different human tumour xenograft models, MDA-MB-231 and PC3. MDA-MB-231 breast cancer and PC3 prostate cancer were selected for in vivo testing of nfP2X7-targeting CAR-T cells as these cell lines represent two of the most common types of cancer and have been widely studied as preclinical models of human tumour xenografts^24–30^. Prior to injection, untransduced and CAR-T cells displayed similar activation, cytotoxicity and co-inhibitory molecule phenotypes (Supplementary Figs. 6a, b, 7a–c). Intravenous administration of 2 × 10^7^ nfP2X7-M CAR-T cells on day 3 post-inoculation with MDA-MB-231 cells significantly inhibited tumour growth in the NSG mice. This was reflected in both tumour size at d39 post-tumour injection and tumour weights at d40 post-tumour injection; with tumour sizes and weights of 59.78 ± 3.27 mm^2^, 267.79 ± 24.89 mg in mice receiving untransduced T cells and 32.44 ± 2.27 mm^2^, 133.55 ± 18.69 mg in mice receiving CAR-T cells (mean tumour size ± SEM, Fig. 6a) Ex vivo analysis of tumours at endpoint recovered significantly higher frequencies of human CD3^+^ T cells in mice administered nfP2X7-M CAR-T cells compared to untransduced control cells (Fig. 6b). Furthermore, there was an increase in the CD4^+^:CD8^+^ T cell ratio in the intratumoural CAR-T cell population with a ratio of 3.4:1 compared to 1.1:1 for recovered untransduced cells (Fig. 6b). T cell subsets were assessed by CD45RO and CD62L expression, which identified the majority of intratumoural cells in mice receiving nfP2X7-M CAR-T cells as CD45RO^+^ CD62L^-^ T_EM_ (89.85 ± 1.97% of CD4^+^, 64.47 ± 5.13% of CD8^+^) with low frequencies of CD45RO^+^ CD62L^+^ T_CM_ and CD45RO^-^ CD62L^-^ T_EFF_ cells also present (Fig. 6c). This was accompanied by a marked increase in the frequencies of co-inhibitory receptor expression on ex vivo recovered T cells. In the CD4^+^ compartment of T cells recovered from CAR-T cell-treated mice, 90.82 ± 1.39% and 55.74 ± 2.55% were found to be PD-1^+^ and CTLA-4^+^, respectively. In the CD8^+^ compartment, 14.42 ± 1.55% expressed CTLA-4 and variable expression of PD-1 ranging from 6.72-86.80% was detected. Furthermore, LAG-3 was expressed by 17.65 ± 2.68% and 51.51 ± 4.38% of CD4^+^ and CD8^+^ T cells, respectively (Fig. 6d). The dramatic phenotypic changes observed in the intratumoural CAR-T cell population compared with their pre-injection phenotype (Supplementary Fig. 6) indicate CAR-T cells likely form antigen-specific interactions with tumour cells and are chronically activated in the tumour microenvironment.

**Fig. 7:**
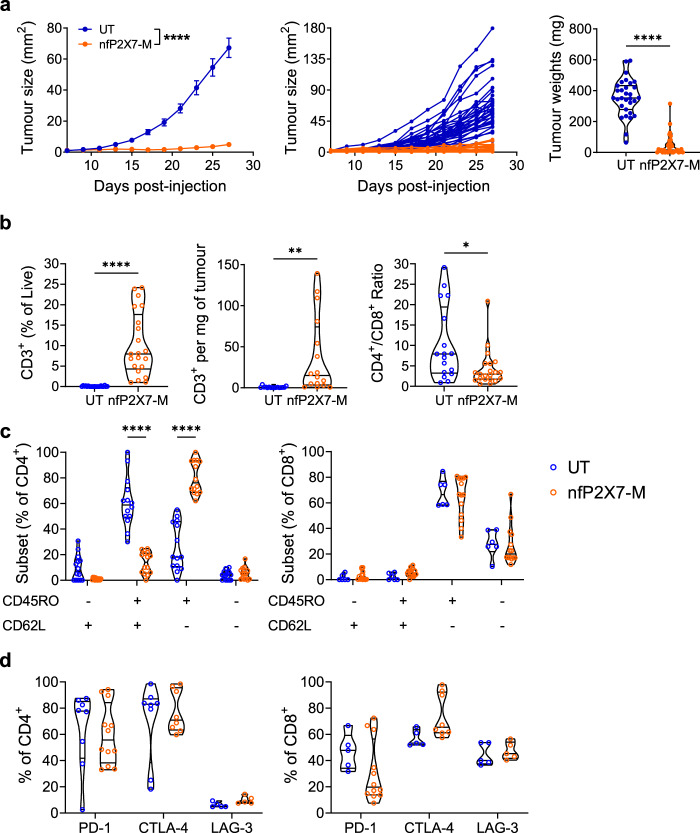
NfP2X7-targeting CAR-T cells significantly inhibit the tumourigenesis of a human prostate cancer xenograft model. 6–8-week-old male NOD-*scid* IL2Rγ^null^ (NSG) mice were subcutaneously injected with 1 × 10^6^ PC3 human prostate cancer cells into the lower abdomen and intravenously injected with 2 × 10^7^ nfP2X7-targeting CAR-T cells or untransduced T cells on d3 or d7 post-tumour injection. Tumours were harvested for flow cytometric analysis at d27-31 post-tumour injection. **a** Tumour growth curves (as pooled and individual mice); *n* = 33 (UT) and *n* = 35 (nfP2X7-M) and endpoint tumour weights; *n* = 31 (UT) and *n* = 34 (nfP2X7-M). Tumour size: two-way ANOVA with Bonferroni’s post-test, ****p < 0.0001; tumour weight: two-tailed unpaired *t-*test, ****p < 0.0001. **b** Frequency of human CD3^+^ T cells of total viable cells in tumours (*n* = 21 (UT) and *n* = 21 (nfP2X7-M)), number of human CD3^+^ T cells per mg of tumour (*n* = 16 (UT) and *n* = 16 (nfP2X7-M)) and CD4^+^/CD8^+^ ratio of CD3^+^ (*n* = 17 (UT) and *n* = 21 (nfP2X7-M)); two-tailed unpaired *t-*test, ****p < 0.0001, **p = 0.0035, *p = 0.0104. **c** Frequencies of T cell subsets as defined by CD45RO and CD62L expression by intratumoural CD4^+^ and CD8^+^ T cells; *n* = at least 6 (UT) and *n* = 14 (nfP2X7-M); two-way ANOVA with Bonferroni’s post-test, ****p < 0.0001. **d** Frequency of PD-1, CTLA-4 and LAG-3 expression by intratumoural CD4^+^ and CD8^+^ T cells; *n* = 8 (UT CD4^+^ PD-1, CTLA-4), *n* = 5 (UT CD4^+^ LAG-3; UT CD8^+^ PD-1, CTLA-4, LAG-3) and *n* = 12 (nfP2X7-M CD4^+^/CD8^+^ PD-1), 8 (nfP2X7-M CD4^+^/CD8^+^ CTLA-4), 5 (nfP2X7-M CD4^+^/CD8^+^ LAG-3). Data in (**a**) are pooled from 6 independent experiments and in (**b**–**d**) pooled from at least 3 independent experiments. Data pooled from in vivo delivery of 5 independent CAR-T cell preparations derived from 4 healthy donors. Data represented as mean ± SEM.

To further explore the potential tumour targeting of nfP2X7-M CAR-T cells, we tested a second human solid tumour model. In this model, NSG mice were injected subcutaneously with PC3 prostate cancer cells and 3 to 7 days later, were administered with a single dose of 2 × 10^7^ nfP2X7-M CAR-T cells via the intravenous route. This resulted in highly significant inhibition of tumour growth (mean size ± SEM 4.90 ± 1.11 mm^2^) compared to mice receiving untransduced T cells (67.23 ± 6.30 mm^2^) at d27 post-tumour injection (Fig. 7a). This was also reflected in the final tumour weight (CAR-T: 43.27 ± 11.23, untransduced: 356.30 ± 21.39 mg) (Fig. 7a, right panel). Similar to MDA-MB-231 tumours, nfP2X7-M CAR-T cells were significantly enriched in tumours compared with untransduced CD3^+^ T cells, with human CD3^+^ T cells comprising 10.27 ± 1.62% of total live cells in tumours of nfP2X7-M CAR-T-injected mice. This is in contrast to the extremely low to undetectable infiltration of untransduced cells in control mice (Fig. 7b, left and centre panels). In this model, the CD4^+^:CD8^+^ ratio of recovered CAR-T cells was 4.3:1 (Fig. 7b, right panel). This increased proportion of CD4^+^ T cells recovered from tumours was consistent with the results observed in the MDA-MB-231 breast cancer model. Similar to the MDA-MB-231 model, the majority of recovered T cells from mice receiving nfP2X7-M CAR-T cells in the PC3 model were CD45RO^+^ CD62L^-^ T_EM_ (80.31 ± 3.42% of CD4^+^, 64.44 ± 4.33% of CD8^+^) (Fig. 7c). and co-inhibitory receptor expression increased significantly ex vivo in both the CD4^+^ compartment (59.86 ± 6.58% PD-1^+^ and 77.17 ± 5.69% CTLA-4^+^) and the CD8^+^ compartment (31.09 ± 6.73% PD-1^+^ and 74.04 ± 5.87% CTLA-4^+^) (Fig. 7d). In comparison, less than 5.64% and 7.61% of input cells expressed CTLA-4 and PD-1, respectively (Supplementary Fig. 6b). Furthermore, LAG-3 was expressed on 9.78 ± 1.34% of the CD4^+^ compartment and 47.40 ± 2.96% of the CD8^+^ compartment of ex vivo recovered T cells. Together, these data suggest a high level of activation in the tumour microenvironment (Fig. 7d). Notably, in one experiment, 2 × 10^7^ nfP2X7-targeting CAR-T cells led to rapid tumour rejection with a complete loss of palpable tumours 18 days post-T cell transfer. These mice exhibited significantly prolonged survival (90 days post-tumour injection) compared with mice receiving PBS or untransduced CD3^+^ T cells (Supplementary Fig. 11).

## Discussion

CAR-T cell immunotherapy holds great promise in the treatment of cancer. However, to date, success has been limited to haematological malignancies. While there are a number of CAR-T cell therapies currently undergoing early clinical trials for treatment of solid tumours, many challenges remain, including selection of tumour-specific antigen targets and identification of the optimal phenotype of CAR-T cells required for effective eradication of solid tumours. Moreover, the generation of novel CARs with anti-tumour efficacy against a broad range of solid tumours represents an attractive strategy. In the present study, we investigated the non-functional form of the purinergic receptor P2X7, nfP2X7, as a candidate target antigen for CAR-T cell therapy, based on observations that the non-functional form is exclusively expressed on cancers and is antigenically distinct from the functional form found on healthy tissues^12,14,31^. Several therapeutic advantages are presented by generating an nfP2X7-specific CAR. The nfP2X7 antigen is expressed on multiple cancer types, and is a unique cancer-specific epitope, rather than an endogenous antigen overexpressed on cancer cells. This raises the possibility of generating a pan-cancer CAR-T cell therapy with minimal on-target off-cancer effects.

We used the binding region from a published peptide binder engineered from a heavy chain variable domain that had undergone multiple rounds of random mutagenesis and phage display affinity purification^17^ to design the nfP2X7-specific CAR binding domain and established gene delivery and cell culture protocols for generation of sufficient number of CAR-T cells with a favourable T cell phenotype for validation of efficacy. To optimize CAR-T cell target recognition, we investigated the effect of different hinge region lengths of the CAR. As previously described, the optimal hinge length is known to vary depending on the target antigen and the relative position of the epitope on the cell surface^21^. This dependence on hinge length is likely due to the need to form an optimal immunological synapse for T cell activation^21,32^. In addition, the hinge region can influence CAR-T cell phenotype and migration capacity independently of tumour antigen binding^33^. Our data confirm that hinge length plays an important role in determining the activity of nfP2X7-targeting CAR T cells, as only T cells expressing a CAR with the medium (IgG4 hinge-CH3) linker demonstrated cytokine release and cytotoxicity against the cancer cell lines tested in vitro. This result does not appear to be due to a difference in surface CAR expression levels, as the frequency of EGFRt reporter expression of the medium hinge length (nfP2X7-M) CAR was not significantly different to short (nfP2X7-S) and long (nfP2X7-L) hinge length CAR-T cells. Given that all three CARs express the same binding domain, but only the medium length hinge induces potent cytotoxicity, we infer this specific hinge forms the required spatial interaction with the epitope, resulting in T cell activation via the intracellular domains. These results clearly demonstrate the importance of testing CARs with a library of hinge lengths to identify the optimal CAR formulation for activity.

As initial CAR-T cell growth in recombinant human IL-2 resulted in sub-optimal expansion and favoured a terminally differentiated CAR-T cell phenotype, further optimisation of the manufacture process involved including IL-7 and IL-15 in combination with IL-2. This cytokine cocktail was utilized during CAR-T cell rapid expansion as multiple studies have shown that IL-7 and IL-15 preferentially expand memory sub-populations such as T_CM_ and T_SCM_ subset phenotypes^34–38^. While there was minimal impact on maturation markers, this combination of IL-2, IL-7 and IL-15 facilitated expansion to higher cell yields (Supplementary Fig. 12) and was consequently adopted as the standard expansion cocktail. Whilst our initial screening to identify the lead CAR-T cell formulation was performed using purified CD8^+^ T cells, we pivoted to generate CAR-T cells from a CD3^+^ T cell pool. This gave the advantage of generating a mixed CD4^+^ and CD8^+^ CAR-T cell population from a single transduction event, potentially simplifying manufacturing and reducing the cost of production of the therapy. Furthermore, CD4^+^ T cells display key advantages for adoptive T cell therapy. For instance, CD4^+^ T cells have been demonstrated to directly lyse tumour cells, possess enhanced in vivo persistence relative to CD8^+^ T cells and in some models, elicit superior anti-tumour activity compared with CD8^+^ T cells^39–42^. In addition, many reports have shown that mixed CD4^+^/CD8^+^ CAR-T populations are more effective in controlling in vivo tumour growth than CD8^+^ CAR-T cells administered alone^43,44^.

A key requirement for clinical translation of CAR-T cell therapy is robust expansion of CAR-T cells that retain desirable phenotypic attributes, such as naive and memory subset phenotypes that possess enhanced self-renewal capacity and are favourable for long-term protective immune surveillance to prevent relapse^45,46^. By testing a range of different culture conditions and using a combination of T cell growth factors, we were successful in generating a CAR-T cell pool comprising a mix of T_N_, T_CM_, T_EFF_ and T_EMRA_ subset phenotypes. Importantly, approximately 30–40% of the CD45RA^+^ CD62L^+^ naive population displayed a T_SCM_ phenotype, which were defined as CD45RA^+^, CD62L^+^, CCR7^+^ and CD95^+^ in line with previously published studies^47,48^. These cells possess the ability to self-renew, are highly proliferative, differentiate into memory and effector phenotypes and possess superior persistence and anti-tumour effects in many cancer immunotherapy models when compared with other T cell subsets^35,46,47,49–52^. We generated a low percentage of effector memory cells consistent with clinically efficient CAR-T cell populations^53^. Although effector T cells possess potent cytotoxicity ability in vitro, they have limited proliferation capacity and persistence in vivo and express higher levels of exhaustion-associated markers^34,54^. Hence the combination of T_N_, T_SCM_ and T_CM_ T cell subsets may provide the most durable anti-tumour protection. Furthermore, the low expression of multiple co-inhibitory molecules associated with T cell dysfunction such as PD-1, CTLA-4 and LAG-3, as observed in the optimised nfP2X7-M CAR-T cells generated in the present study, has been correlated with increased anti-tumour efficacy^55^. Using our current expansion protocol, we observed TIM-3 expression on a large percentage (>70%) of untransduced and CAR-T cells in both the CD4^+^ and CD8^+^ compartments. The role of TIM-3 as a co-inhibitory or co-stimulatory receptor is somewhat controversial. High frequencies of TIM-3^+^ in CAR-T cell populations has also been reported by several other groups^56–59^. However, the expression of TIM-3 does not seem to impair CAR-T cell in vivo anti-tumour efficacy as demonstrated by the work described here and by others^56,59^. Furthermore, expression of TIM-3 without PD-1, LAG-3 or CTLA-4 on the majority of CAR-T cells suggest these cells are not highly dysfunctional^60–63^.

A major advantage of the nfP2X7-M CAR is its potential broad specificity against a wide range of cancers. In total, nfP2X7-M CAR-T cells were tested for in vitro cytotoxicity against twenty-four different cell lines derived from twelve cancer types, resulting in varying degrees of cytotoxicity against each of the different cancer cell lines tested. We tested nfP2X7-M CAR-T cells against two triple-negative breast cancer cell lines MDA-MB-231 and BT549. MDA-MB-231 cells are highly aggressive, and model triple-negative breast cancers associated with lower survival rates, while BT549 are considered less aggressive. While significant cytotoxicity was observed against both BT549 and MDA-MB-231 cells, CAR-T cells elicited higher cytotoxicity against BT549 than MDA-MB-231 at all E:T ratios tested. This was accompanied by increased cytolysis of BT549 cells with the untransduced control, suggesting this cell line is more susceptible to non-antigen-specific T cell-mediated killing. Both P2X7 and nfP2X7 are reported to be expressed in ovarian cancer^12,14^. In keeping with this, the nfP2X7-M CAR-T cells showed significant cytotoxicity against two different ovarian cancer cell lines; OVCAR5 and OVCAR3 in vitro.

We observed no cytotoxicity against three cell lines (multiple myeloma RPMI-8226, leukaemia K562 and neuroblastoma SK-ND-Z). Indeed, a separate study demonstrated RPMI-8226 cells are nfP2X7-negative following staining with a monoclonal antibody specific for the E200 epitope of nfP2X7^12^. The lack of cytotoxicity for the K562 and SK-ND-Z is also likely due to decreased antigen density, but as an nfP2X7-specific antibody is currently not commercially available, we were unable to measure nfP2X7 levels in the different cancer cell lines tested in this study. However, our data are consistent with previous antibody-based studies examining nfP2X7 and P2X7 levels and functional status in several cell lines^11,12^. Gilbert et al.^12^ examined P2X7 and nfP2X7 levels in a number of cancer cell lines using ion transport as a measurement of pore function for P2X7 and flow cytometric analysis to assess nfP2X7 expression. This study showed PC3 (prostate) and Kelly (neuroblastoma) cell lines expressed nfP2X7 and low levels of P2X7, whereas SK-MEL-5 (melanoma) expressed high levels of P2X7 and low levels of nfP2X7, highlighting a generally inverse relationship between P2X7 and nfP2X7 surface expression. This is with the exception of the Ramos lymphoma cell line, which showed an absence of both nfP2X7 and P2X7 expression^12^. Our cytotoxicity assays were largely consistent with those observations, with statistically significant cytotoxicity observed against PC3 and Kelly cell lines (60% and 45% cytolysis above the UT control). Furthermore, we could not detect significant activity against the Ramos and SK-MEL-5 cell lines or healthy donor-derived PBMCs, which are reported to express wild-type P2X7. P2X7 was reported to be highly expressed in neuroblastoma primary tumours and cell lines^64^, but we only observed high cytotoxicity against the Be(2)-M17, Kelly and SH-SY5Y cell lines and not SK-ND-Z. Aside from the potential absence of nfP2X7 expression, it is also possible that the SK-ND-Z cells express a different form of nfP2X7, and the epitope targeted by nfP2X7-M CAR-T cells is not present on this non-functional form of the receptor. Furthermore, point mutations, SNPs, splice variants and post-translational modifications of P2X7 that impair pore formation have been reported^12,15^ and specific mutations of P2X7 are known to be positively selected on transformed cells^65^. However, based on those previous studies overall, it is likely that the variation in anti-tumour efficacies observed in the present study reflects differences in antigen density and/or heterogeneity of nfP2X7 expression under in vitro culture conditions. To unequivocally demonstrate the on-target specificity of nfP2X7-targeting CAR-T cells, we used CRISPR Cas9 to target the endogenous *P2X7* locus in the prostate cancer cell line PC3. Having confirmed both alleles of the gene had been targeted, we showed a statistically significant reduction in specific killing of this PC3 clone by nfP2X7-M CAR-T cells compared with negative control PC3 cells transduced with a non-targeting sgRNA, which supports the mechanism of action being dependent on expression of the *P2X7* gene. Taken together, the results from these in vitro experiments combined with previous studies which reported nfP2X7 expression on a diverse range of malignant cells indicate that nfP2X7-M CAR T cells are broad-acting and specifically respond to nfP2X7 but not wild-type P2X7 on the cell surface.

Encouraged by the cytotoxic activity against multiple cancer cell lines in vitro, we next assessed the clinical utility of nfP2X7-targeting CAR-T cells in preclinical in vivo modelling using two well-established xenograft models. These in vivo models are critical for demonstrating a path to clinical utility as they impose a significant selection pressure on the CAR-T cell trafficking to the tumour and binding to the target epitope on the cancer cells. To test the in vivo anti-tumour efficacy of nfP2X7 CAR-T cells, we utilized the MDA-MB-231 breast cancer and PC3 prostate cancer xenograft models, as these cancers represent two of the most common types of solid tumours. MDA-MB-231 cells display a triple-negative, highly aggressive phenotype, which recapitulates late-stage breast cancer and preferentially metastasizes to the lymph nodes and lungs^66–68^. NfP2X7 has been reported to be expressed on malignant cells in breast cancer patients^12^. Furthermore, it has been reported that human prostate cancer biopsies express the nfP2X7 receptor and an increase in expression is associated with cancer progression^31,69^. In the present study, preclinical testing of nfP2X7-targeting CAR-T cells demonstrated significant inhibition of tumour growth of both human breast cancer and prostate cancer models.

In six independent experiments in the PC3 prostate cancer xenograft model, we consistently observed significant tumour inhibition in mice administered nfP2X7-targeting CAR-T cells compared to mice administered untransduced T cells. In one experiment, we observed 100% survival of nfP2X7-M CAR-T cell-treated mice up to 90 days post-tumour inoculation. To our knowledge, our study shows the first instance of CAR-T cells independently inducing complete rejection in the PC3 prostate xenograft model using unmodified tumour cells which were not engineered to overexpress the CAR-targeting antigen^29,30,70^. We did not routinely track tumour growth long-term in the in vivo experiments, as our primary rationale was to harvest tumours once euthanasia criteria was reached for any of the groups (normally the control PBS and/or untransduced groups) to allow for parallel comparison of ex vivo phenotypes. The efficacy in the PC3 model was associated with significant CAR-T cell intratumoural infiltration. A similar but less potent effect on tumour inhibition was observed in the MDA-MB-231 breast cancer xenograft model. To date, most preclinical studies involving transfer of CAR-T cells into a human triple-negative breast cancer models utilize MDA-MB-231 cells. Indeed, separate studies which have administered CAR-T cells into the MDA-MB-231 model demonstrate only modest tumour inhibition^25–27^. In addition, the number of such studies is limited and it is important to note that none of the studies demonstrated complete rejection of MDA-MB-231 tumours and they often did not follow survival of the mice^66–68^.

In the results presented in this study, the majority of tumour-infiltrating CAR-T cells were of a T_EM_ or T_EFF_ subset phenotype and acquired the expression of co-inhibitory receptors. This enhanced co-inhibitory molecule expression following ex vivo recovery may be indicative of the T cells receiving persistent stimulation in the tumour microenvironment, resulting in rapid proliferation and upregulation of these markers by the experimental endpoint (24–37 days post-T cell transfer). While co-inhibitory molecules act to curtail T cell responses, paradoxically these same molecules also represent T cell activation markers. PD-1 in particular has been re-contextualised as a marker of activated tumour-reactive T cells^71,72^. Phenotypic changes were more evident in the CD4^+^ compartment, which was associated with an increased proportion of CD4^+^ cells recovered from tumours compared to the CD4^+^/CD8^+^ ratio at pre-injection. Indeed, this increased frequency may be attributed to enhanced proliferation and activation of CD4^+^ cells. Interestingly, some studies have reported enhanced persistence of tumour-specific CD4^+^ cells compared to CD8^+^ cells following antigen stimulation, due to a lowered propensity to activation-induced cell death and bona fide exhaustion^40^. While co-inhibitory molecule expression was also significantly increased on CD8^+^ T cell populations compared to at pre-injection, the relative frequencies were lower than CD4^+^ T cells, which may be due to increased turnover of activated and highly tumour-reactive CD8^+^ T cells.

The observed differences in efficacy between the MDA-MB-231 and PC3 models could be due to a number of factors, including differences in levels of antigen expression, differences in the percentage of tumour cells expressing the antigen, or differences in the ability of the tumours and the associated tumour microenvironment to limit CAR-T cell recruitment or suppress CAR-T cell function. From our in vitro data, it appears that PC3 cells are more susceptible to nfP2X7-M CAR-T cell-mediated cytotoxicity than MDA-MB-231 cells, potentially due to a higher level of antigen expression per cell or a greater frequency of cells expressing sufficient antigen. Loss of antigen expression has been reported as a mechanism for the development of resistance to the CAR-T cell treatment^73^ and loss of nfP2X7 expression, which may have occurred in MDA-MB-231 tumours, may have also contributed to the differences in tumour susceptibility to nfP2X7-targeting CAR-T cells in vivo. While this research paves the way for initial clinical studies in humans, the availability of a high specificity nfP2X7-specific monoclonal antibody is crucial in order to screen patients for tumour expression of nfP2X7 to identify those patients who will most likely benefit from nfP2X7-targeting CAR-T cell therapy.

In summary, our results clearly highlight the potential of nfP2X7-targeting CAR-T cells as a novel immunotherapy for multiple solid cancer types. This study is the first to demonstrate that nfP2X7 has great potential as a cellular immunotherapy target for a broad range of nfP2X7-expressing solid tumours, and that the CAR-T cell modality may provide a functional alternative to targeting nfP2X7 with a monoclonal antibody^16^. Indeed, it is rare to see tumour rejection or cure in NSG mice in CAR-T cell studies against solid tumours where mice only receive CAR-T cells with no additional treatment (e.g. checkpoint inhibitors, radiation, IL-2 administration) or where there is no modification of tumour cells. Further approaches to improve the clinical efficacy of nfP2X7-targeting CAR-T cells may involve administration in combination with standard surgical and/or chemotherapy regimens, or with checkpoint inhibitors.

The efficacy of nfP2X7-targeting CAR-T cells, either alone or in combination with other therapies will need to be tested in clinical trials after stratification of participants with nfP2X7-positive tumours. Despite this, results presented in this study position nfP2X7 as a promising target for CAR-T cell therapy, overcoming a major challenge in the identification of a suitable target antigen for immunotherapy and providing a potential avenue to translate the success of CAR-T cell therapies from haematological malignancies to a broad range of solid cancers.

## Methods

### Cancer cell lines and primary cells

Details of all cell lines used in this study are listed in Supplementary Table 1. For in vitro cytotoxicity assays, cancer cell lines were transduced with a lentiviral vector encoding the genes for luciferase and green fluorescence protein (GFP) separated by the self-cleavage peptide T2A. Human CD3^+^ T cells were isolated from whole blood or buffy coats obtained from anonymous healthy donors under consent (SSA/19/WCHN/96)(HEC/19/WCHN/65).

### Generation of P2X7 knockout clones

PC3-luciferase cells were seeded at 1.2 × 10^5^ cells per well of a 12-well plate and incubated overnight to attach. The following day, the cells were transfected with synthetic guide RNAs (P2X7guide4; AUUAGUGACGGGGGGAACCG, P2X7guide5; ACUACACCACGUAAGUGCCC, P2X7guide6; CUCGGAAGAUGUCUCCUAGU and P2X7guide7; CACAGGAGAAACAUCCUGCC, CRISPRevolution sgRNAs SYNTHEGO Corporation Redwood City, CA). Guide sequences were selected from a ranked list of potential SpCas9 cleavage sites in the human genome (GRCh38/hg38, CRISPR/Cas9-NGG Targets; https://genome.ucsc.edu/). A negative control guide #1 was also purchased from SYNTHEGO. PC3 cells were transfected with SpCas9 2NLS Nuclease/gRNA ribonucleoprotein (RNP) complexes with Lipofectamine™ CRISPRMAX™ Transfection Reagent (ThermoFisher) as per the manufacturer’s protocol with the following modification. The RNP complex was formed by incubating equimolar amounts of the four P2X7 guides (7.8pmol/guide) with SpCas9 2NLS Nuclease (6pmoles). RNP complexes containing the negative control guide was also formed with 7.8 pmol sgRNA per 6pmol of SpCas9. Cells were passaged three days post-RNP transfection and genomic DNA was isolated using QuickExtract™ DNA Extraction Solution (Lucigen). Deletion of the target region was determined by PCR using AmpliTaq Gold 360 master mix (ThermoFisher) and 100 μM each of Forward GCTTTGCCCACTAGGTTTGC and Reverse CCACCAACCTGAATTGCCAC primers and an annealing temperature of 58 °C. PCR products corresponding to putative non-deleted (986 bp) and deleted alleles (160–397 bp) were gel purified using a NucleoSpin® Gel and PCR Clean-up kit (MACHEREY-NAGEL) and subjected to Sanger sequencing to confirm their identity. Individual PC3-Luc clones from the knockout and negative control pools were then created by limiting dilution (100 μl/well of 10 cells/ml) in 96-well plates. After 3 weeks in culture, positive wells were scored for single colonies and 42 individual clones from the P2X7 knockout pool were screened by PCR. Characterisation of the specific guide driven mutagenesis at the P2X7 alleles of the identified knockout clone 21 was determined by gel purifying the individual PCR products and cloning these into the pCR4-TOPO-TA vector using a TOPO™ TA Cloning™ Kit (ThermoFisher) followed by Sanger sequencing (M13 forward and reverse primers), as shown in Fig. 3.

### CAR construct design

The nfP2X7 binding domain^12,15–17^ was cloned into a well characterised second-generation CAR vector backbone^19,54^ which encoded the hinge/linker region, CD28-derived transmembrane domain, intracellular signalling domains 41BB and CD3 zeta, self-cleavage peptide T2A and a truncated form of the EGFR receptor (EGFRt)^19,74^. We tested three hinge lengths short (nfP2X7-S: IgG4 hinge), medium (nfP2X7-M: IgG4 hinge-CH3) and long (nfP2X7-L: IgG4 hinge-CH2-CH3).

### Manufacture of lentivirus

Lentivirus was produced by transfecting HEK 293T/17 cells (ATCC No-CRL11268) with the third-generation self-inactivating lentiviral plasmid and the packaging plasmids encoding pCMV-Rev, pCMV-VSV-G and psPAX2 (gag-pol), using the manufacturer’s protocol for Lipofectamine 3000 (Thermofisher Scientific). Supernatants were collected 24 h and 48 h post-transfection and concentrated by ultracentrifugation at 50,000 × *g* for 2 h at 4 °C. The pellet was resuspended in Opti-MEM reduced serum media (Life Technologies) and stored at −80 °C. Virus titres were determined by transduction of 1 × 10^5^ HEK293T-17 or Jurkat cells with serial dilutions from 1:100 to 1:2000 of the concentrated virus in the presence of 8ug/ml polybrene (Sigma-Aldrich), followed by analysis of EGFR reporter expression via flow cytometry. Titres were calculated based on reporter expression at 48 h^75,76^.

### T cell transduction and expansion

CD3^+^ T cells were isolated from peripheral blood using the RosetteSep Human HLA T cell enrichment cocktail (StemCell) following the manufacturer’s protocol. Cells were cultured in either complete X-VIVO^TM^ 15 Haematopoietic cell media (Lonza) supplemented with 2 mM HEPES (Gibco) and 5% human serum (Sigma-Aldrich) or complete X-VIVO^TM^ 15 Haematopoietic cell media supplemented with 2% KnockOut serum replacement (Gibco). Culture media was supplemented with the following cytokines (rhIL-2 50 IU/ml, rhIL-7 5 ng/ml, rhIL-15 0.5 ng/ml) (Lonza or Miltenyi Biotec). CD3^+^ T cells were stimulated with α-CD3/α-CD28 Dynabeads (Invitrogen), then transduced with lentivirus in the presence of 8 µg/ml polybrene. Beads were removed and transduction efficiency was determined on day 7 post-transduction. Transduced cells were stained with anti-EGFR monoclonal antibody (me1B3) (eBiosciences) and surface expression was analysed by flow cytometry. On day 8 post-isolation, cultures were further expanded by re-stimulation with peripheral blood mononuclear cells (PBMC). PBMCs were isolated using Ficoll Paque Plus (Sigma-Aldrich) or Ficoll-Paque Premium (GE Healthcare) density gradient centrifugation from buffy coats (50 ml) obtained from LifeBlood (Adelaide) and frozen. Isolated PBMC were thawed and inactivated by irradiation on the day of T cell rapid expansion. Inactivated PBMC were added to the T cell cultures, at a T cell to PBMC ratio of up to 1:50 in the presence of a soluble α-CD3 antibody (30 ng/ml, OKT3) (Invitrogen). Cells were cultured for 12–14 days in G-Rex6® well plates (Wilson Wolf), with appropriate media changes and cytokine additions every 2–3 days. Cell counts and cell phenotype analysis were performed 12–14 days post-irradiated PBMC co-culture. Cells were routinely frozen on d15 post-PBMC for subsequent in vivo delivery.

### Design of a nfP2X7 peptide mimetic for flow cytometry

To detect CAR surface expression and epitope-specific binding, a biotin-labelled peptide was designed and synthesised (GenScript). This peptide consisted of a biotin moiety at the N terminus attached to the 16 amino acid sequence of P2X7 recognised by the CAR with a four amino acid (SGSG) linker.

### Immuno-phenotyping of CAR-T cells using flow cytometry

Four different antibody panels were used to assess CAR-T cell phenotype. These included surface markers used to define the stage of cell maturation (CD45RA, CD45RO, CD62L and CCR7), cytotoxicity markers (IFN-ɣ, TNF-α, IL-2, Granzyme B, Perforin and CD107a), activation markers (CD27, CD28, CD95, CXCR3) and co-inhibitory receptors/molecules (PD-1, CTLA-4, LAG-3, TIM-3, CD39). Antibodies are listed in Supplementary Table 2.

Cultured T cells (1 × 10^5^) or ex vivo single-cell tumour suspensions (1 × 10^6^) were stained in 96-well round-bottom plates (Corning), using antibodies and related reagents detailed in Supplementary Table 2. For intracellular cytokine and cytotoxic molecule staining, cells were first incubated at 37 °C, 5% CO_2_ for 4 h in complete IMDM (Gibco) supplemented with 10% FCS (Sigma-Aldrich), 100 U/ml penicillin/streptomycin (Gibco), 1x GlutaMAX (Gibco), 54pM β-mercaptoethanol (Sigma-Aldrich), 50 ng/ml phorbol 12-myristate 13-acetate (Life Technologies), 1 nM ionomycin (Life Technologies), 1/1500 GolgiStop (BD Biosciences) and 1/1000 GolgiPlug (BD Biosciences or Biolegend). To stain CD107a, a directly conjugated antibody was added at the beginning of the PMA stimulation. All subsequent incubations were performed at room temperature unless stated otherwise. Cells were washed in PBS, before being stained with Fixable Viability Stain 780 (BD Biosciences) diluted 1/1000 and blocked with Human FC Block (BD Biosciences) for 10 min. Cells were then washed in FACS buffer (PBS 1% BSA, 0.04% azide) and stained for 30 min with directly conjugated antibodies in Brilliant Stain Buffer (BD Biosciences). For intracellular staining, cells were incubated with Cytofix/Cytoperm (BD Biosciences) for 20 min at 4 °C, washed in Perm/Wash buffer (BD Biosciences) and stained with intracellular directly conjugated antibodies for 30 min at 4 °C. For the biotinylated nfP2X7 peptide mimetic, cells were stained with the peptide mimetic along with directly conjugated antibodies at room temperature, washed in FACS buffer and stained with streptavidin conjugated to BV421 (BD Biosciences) for 20 min at 4 °C. After staining, cells were washed once with FACS buffer, washed once with PBS or PBS 0.04% sodium azide, resuspended in PBS 1% paraformaldehyde and stored at 4 °C in the dark. Stained cells were acquired on the BD LSRFortessa X-20 flow cytometer within 5 days. Beckton Dickinson FacsDiva software was used for Flow cytometry raw data acquisition. Data analysis was performed using FlowJo Software V.10 (TreeStar, BD Biosciences). Gating strategies are detailed in Supplementary Figs. 1–5.

### Cytokine release assay

Activation of CAR-T cells was measured by cytokine release assays. Target cell lines were co-cultured with CD4^+^ T cells (control untransduced or CAR-T cells) for 24 h at 37 °C and the concentration of cytokines; IL-2, IFN-ɣ and TNF-α in the supernatant was assayed using a Bio-Plex (Bio-Rad) validation kit.

### In vitro cytotoxicity analysis

Two methods were used to assess cytotoxic activity of CAR-T cells in this study. Cytotoxicity was measured using a standard 4 h ^51^Cr release assay^77^ using the following effector: target cell (E:T) ratios; 30:1, 10:1, 3.3:1, 1.1:1. Briefly, target cells were labelled with ^51^Cr (5 mCi/ml) overnight at 37 °C with 5% CO_2_, and on the next day cells were washed with PBS and plated (5 × 10^3^ cells/well in 100 µl) in 96-well plates. Effector cells were then added to the target cells at different ratios in triplicate in a total volume of 100 µl and were co-cultured for 4 h at 37 °C with 5% CO_2_. Additional control wells with target cells alone were plated for each target cell line. To determine the maximum cytotoxicity, 100 µl of 2% SDS solution was added to target cells alone. To determine the minimum cytotoxicity or assay background level, 100 µl of media (RPMI media supplemented with 10% FCS) was plated. After 4 h incubation, 50 µl of the supernatant was harvested and transferred to white LUMA plates. The LUMA plates were air-dried overnight and read using a TopCount scintillation counter (Perkin Elmer). Percentage cytotoxicity was calculated as (CPMsample − CPMMin)/(CPMMax − CPMMin) × 100.

To replace the ^51^Cr radioactive CTL assay with a safe non-radioactive equivalent, the Bright-Glo luciferase assay system (Promega) was used to determine the cytotoxicity of cancer cell lines by CAR-T cells. Cancer cell lines stably expressing luciferase were used as target cells and seeded (1 × 10^4^ in 50 µl) into a round bottom 96-well plate in triplicate for each condition tested. CAR-T cells (50 µl) were added to the target cells in the following effector: target ratios (10:1, 3:1, 1:1). Additional control wells of media alone and target cells alone were included to determine minimum luminescence and maximum luminescence, respectively. Cells were incubated for 16 h at 37 °C with 5% CO_2_ prior to the addition of Bright-Glo assay substrate (100 µl) (Promega) and further incubation for 4 min at room temperature. An aliquot (100 µl) of the mix was then transferred to an 96-well opaque plate (Nunc) and luminescence measured using a luminometer (GloMax Promega). Percentage cytotoxicity was calculated using the following formula: 100 − [(Luminescence sample − luminescence media alone)/(Luminescence target alone − luminescence media alone) × 100%]. Data analysis was performed using GraphPad PRISM version 9.0.0.

### xCELLigence real-time impedance/cytotoxicity assay

E-plates (ACEA, Biosciences, San Diego, California, USA) were equilibrated in the xCELLigence® RTCA DP instrument (ACEA Biosciences) for 30 min with target cell media prior to the addition of target cells (2.4–3.0 × 10^4^ cells in 100 µl of media). Impedance readings were taken every 15 min up to 16 h to establish a baseline and stabilised impedance. For measuring CAR-T cell cytotoxicity, target cell media (50 µl) was removed and effector cells were added at an E:T of 3:1 in 50 µl of target cell media. Assays were performed in duplicates with impedance readings taken every 15 min for 72 h. For analysis, the cell indices were normalised to the time point when the effector T cells were added. The area of the curves of the plots 72 h post-T cell addition were measured and normalised to the untreated target cells as an indication of T cell killing efficacy.

### In vitro cytotoxicity imaging

Target MDA-MB-231-LM2-GFP cells (a derivative of MDA-MB-231 cells, which were transduced to co-express GFP)^78^ were seeded at 5 × 10^4^ cells in 300 µl target cell media per well of an ibiTreat 8 well µ-slide (ibidi, Martinsried) and allowed to settle and spread overnight. CD3^+^ T cells were prepared at an E:T of 1:1 and pulsed with 5 µM Fluo-4 AM (Invitrogen) at 37 °C for 20 min. The pulsed T cells were washed, resuspended in 200 µl target cell media and added to the respective wells with 200 µM propidium iodide (Invitrogen). Time-lapse images were captured at 10 s intervals for 3 h using a 40x objective lens on the LSM 700 laser scanning confocal microscope (Zeiss) using the Zen 2011 (black edition) software (Zeiss).

### Mice

NOD-*scid* IL2Rγ^null^ (NSG) mice (stock number: 005557) were purchased from the Animal Resource Centre (Perth, WA). Mice were allowed to acclimatise for at least one week upon arrival before experimental procedures were performed. Experiments used gender and age-matched mice between 6–8 weeks of age and experimental and control groups were co-housed. Animal usage for experiments presented in Figs. 6, 7 and Supplementary Fig. 11: Mice were housed at the Commonwealth Scientific and Industrial Research Organisation (CSIRO) facility or Helen Mayo Animal House (HMAH) facility at the University of Adelaide (Adelaide, SA) in specific pathogen-free conditions with a 12 h light/dark cycle. Mice were fed with 2020x Teklad Global Soy Protein-Free Extruded Rodent Diet (Envigo) ad libitum. Mice were humanely euthanized by CO_2_ asphyxiation at the experimental endpoint. Animal usage for experiments presented in Fig. 5: Mice were housed at the University of South Australia Core Animal Facility. Housing: 12-hour light/dark cycle in pathogen-free conditions in sterile IVC cages. Diet: Meat Free Rat and Mouse Diet (Manufacturer, Specialty feed, cat# SF00-100), autoclaved and fed ad libitum. Mice were euthanised by CO2 asphyxiation or cervical dislocation while under anaesthesia (ketamine/xylazine or isoflurane). All experimental procedures were conducted under the approval of the University of Adelaide Animal Ethics Committee (S/2018/007) and University of South Australia Ethics Committee (AEC# U46-19). All experiments conformed to the guidelines established by the ‘Australian Code of Practice for the Care and Use of Animals for Scientific Purposes’.

### Intravital microscopy

Female NSG mice aged 6–8 weeks were purchased from Animal Resources Centre (Perth, Australia). NSG mice received a subcutaneous injection of 2 × 10^6^ MDA-MB-231-LM2 cells, resuspended in a mix of 25 µl cold PBS and 25 µl Matrigel in the mammary fat pad. For intravital microscopy purposes, tumours were allowed to grow for 2 weeks prior to T cell injection. Prior to imaging, tumour-bearing mice were anaesthetised initially with isoflurane/O_2_ before intraperitoneal injection of ketamine/xylazine (10 mg/ml, Ceva Animal Health). The midline of the mouse was then surgically opened, and the skin separated from the abdominal cavity to expose the tumour. nfP2X7-targeting CD3^+^ CAR-T cells or untransduced CD3^+^ control cells (2 × 10^7^ cells in 100 µl of PBS) pre-labelled with CellTracker Orange CMTMR (Invitrogen), together with 50 µl of 50 kDa Cy5 Dextran (Sigma), were injected intravenously through the tail vein. The mouse was then positioned under the LSM710 Two Photon Confocal Laser Scanning Microscope (Zeiss) with the tumour exposed under the objective lens. The tumour was excited with the Mai Tai Ti:Sapphire multiphoton laser (Spectra-Physics, Santa Clara, USA) and external non-descanned detectors used at 780 nm to capture the fluorescence signals to visualise the MDA-MB-231-LM2 cells expressing low levels of GFP, Cy5 Dextran and CellTracker Orange labelled T cells. Time-lapse images were captured at 5 msec intervals under the 20x/1.0 DIC W Plan-Apochomat objective (Zeiss, Carl Zeiss, Jena, Germany) using the Zen 2011 (black edition) software (Zeiss). Animals were humanely killed at the experimental endpoint.

### Tumour tissue histology

Tumours harvested from mice were processed as formalin-fixed paraffin-embedded (FFPE) with 4 µm sections, dewaxed and antigen retrieval performed using Tris-EDTA pH9.0 prior to tissues being blocked with 5% normal goat serum (NGS) in CAS-Block (Life Technologies) for 30 min at room temperature. Primary antibody (anti-CD3 antibody) (1:700; Cell Marque (Sigma-Aldrich)) diluted in CAS-Block plus 5% NGS was added to the tissues and incubated overnight at 4 °C. After washing, secondary antibody Alexa-555 (goat anti-rabbit, 1:500, ThermoFisher Scientific) was prepared in CAS-block with 5% NGS and incubated for 2 h at room temperature. Slides were counterstained with DAPI (1:2000, ThermoFisher Scientific) and imaged using the Zeiss Axio Scan.Z1 slide scanner.

### Xenograft mouse models

Tumour cell lines were cultured according to the conditions detailed in Supplementary Table 1. On the day of injection, adherent cells were rinsed in PBS, incubated with trypsin-EDTA solution (Gibco) at 37 °C, washed once in culture medium and twice in PBS. Cells were kept on ice until injection. NSG mice were anaesthetised using isofluorane (Henry Schein). For the MDA-MB-231 human breast cancer model, 6–8-week-old female NSG mice were injected subcutaneously into the 4th left mammary fat pad (L4) with 2 × 10^6^ MDA-MB-231 cells resuspended in sterile PBS:Matrigel such that the final protein concentration was 4–6 mg/ml. Cells were injected in a 45–50 µl volume using a 26½ gauge needle attached to a 50 µl glass syringe (Hamilton Company, NV, USA). For the PC3 model, 6–8-week-old male NSG mice were injected subcutaneously into the lower abdomen with 1 × 10^6^ PC3 cells resuspended in sterile PBS. Cells were injected in a 200 µl volume using a 26½ gauge needle attached to a 1 ml Luer lock syringe (BD Biosciences). Tumours were measured every 2 days beginning on d7 post-tumour injection using digital callipers (Mitutoyo, Japan) by measuring the longest distance as length and the perpendicular distance as width. Tumour size was calculated as the multiple of length and width measurements. As per ethics requirements (University of Adelaide Animal Ethics Committee (S/2018/007)), the health status of mice was monitored daily and mice were humanely euthanised when the tumours became ulcerated, tumour length was equal to or greater than 15 mm, tumour size was equal or greater than 100 mm^3^ or when mice displayed a combination of disease symptoms including any of the following: ruffled coat, hunched posture, reluctance to move, laboured breathing, weight loss of 10% or more of initial weight and/or changes in behaviour or gait. For intravenous transfer of T cells, CAR-T cells or untransduced T cells were thawed and resuspended in sterile PBS. Mice were restrained and injected with a cell suspension via the tail vein at day 3 or 7 post-tumour injection. Tumour measurements, monitoring and T cell injections were performed with groups blinded.

### Preparation of tumour homogenates

Tumours were excised, manually minced into small pieces and incubated in warm digest media for 1–1.5 h at 37 °C with continuous gentle agitation. Tumours were mixed every 15–20 min by resuspension. Digest media was prepared by supplementing DMEM (Gibco) with 5% heat-inactivated FCS (Sigma-Aldrich), 2.5 mM CaCl_2_, 10 mM HEPES (Gibco), 100 U/ml penicillin/streptomycin (Life Technologies), 30 U/ml DNase I (Sigma-Aldrich) and 1 mg/ml collagenase IA (Sigma-Aldrich). Tumour homogenates were passed through a 70 µm filter (BD Biosciences), washed in PBS and incubated in mouse red cell lysis buffer for 5 min at 37 °C. Cells were then washed in PBS and stained for analysis by flow cytometry.

### Reporting summary

Further information on research design is available in the Nature Portfolio Reporting Summary linked to this article.

### Supplementary information

Supplementary Information

Description of Additional Supplementary Files

Supplementary Movie 1

Supplementary Movie 2

Supplementary Movie 3

Supplementary Movie 4

Reporting Summary

### Source data

Source Data

## Data Availability

The datasets generated during and/or analysed during the current study are available from the corresponding author upon request. Source data are provided with this paper.
