# Supplementary material for: Pre-clinical validation of a pan-cancer CAR-T cell immunotherapy targeting nfP2X7
Source: Nat Commun. 2023 Sep 8;14:5546. doi: 10.1038/s41467-023-41338-y (PMC10491676; doi:10.1038/s41467-023-41338-y)
Supplement: Supplementary file 1 — Supplementary Information [file 41467_2023_41338_MOESM1_ESM.pdf]

Pre-clinical validation of a pan-cancer CAR-T cell immunotherapy targeting nfP2X7.

Supplementary information

V. Bandara[1]*, J. Foeng[2]*, B. Gundsambuu[1], T.S. Norton[2], S. Napoli[1], D.J. McPeake[2], T.S. Tyllis[2], E. Rohani-Rad[2], C Abbott[2], S.J. Mills[3], L.Y. Tan[4], E.J. Thompson[4], V.J. Willet[5], V.M. Nikitaras[5], J. Zheng[1], I. Comerford[2], A. Johnson[6], J. Coombs[7], M.K. Oehler[8], C. Ricciardelli[5], A.J. Cowin[3], C.S. Bonder[4,9], M. Jensen[6], T.J. Sadlon[10], S.R. McColl[2,7]*, S.C. Barry[1,7,10]*#

1 Molecular Immunology RRI University of Adelaide, Adelaide, SA 5000, Australia

2 Chemokine Biology Laboratory, Department of Molecular and Cellular Biology, School of Biological Sciences, University of Adelaide, Adelaide, SA 5005, Australia

3 University of South Australia, STEM (Future Industries Institute) SA, 5095, Australia

4 Centre for Cancer Biology, University of South Australia and SA Pathology, Adelaide, SA 5001, Australia

5 Reproductive Cancer Research group, Discipline Obstetrics and Gynaecology, Robinson Research Institute, University of Adelaide, SA 5005, Australia

6 Seattle Children's Research Institute, WA 98101, United States

7 Carina Biotech, Level 2 Innovation & Collaboration Centre, UniSA Bradley Building, Adelaide SA 5001, Australia

8 Department of Gynaecological Oncology, Royal Adelaide Hospital, SA 5005, Australia

9 Adelaide Medical School, The University of Adelaide, Adelaide, SA 5005, Australia

10 Department of Gastroenterology, Women's and Children's Health Network, North Adelaide, SA 5006, Australia.

*These authors contributed equally

#corresponding Author: simon.barry@adelaide.edu.au

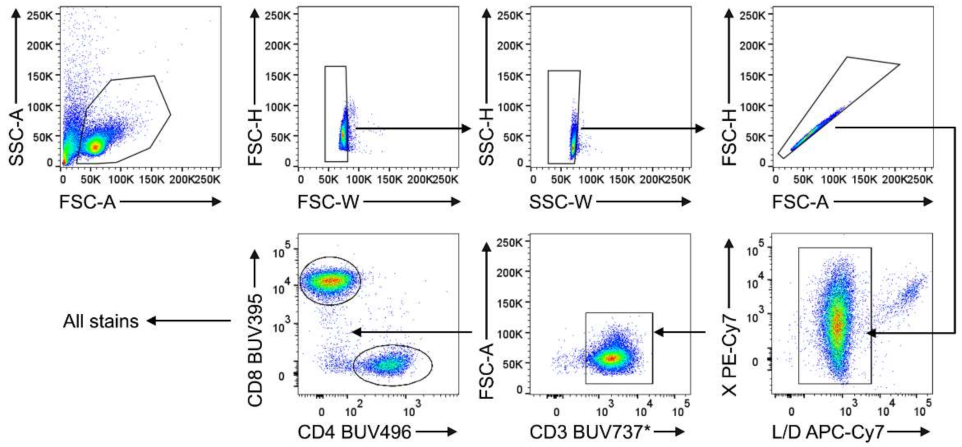

Supplementary Figure 1: Flow cytometry pre-gating strategy for all stains. Lymphocyte gating, single cell discrimination, viability gating, CD3⁺ gating, CD4⁺ or CD8⁺ gating serves as a pre-gate for all flow cytometry data, with the exception of the activation marker phenotyping. *In that panel, CD28-BUV737 was used such that the combination of fluorophores was optimised to reduce spectral spill over. As CD3 was conjugated to the same fluorophore and cells were routinely all CD3⁺, it was not included in the panel. Gating strategy was used for Figures 4b-d and 5b-d. X represents CD95, CD45RO, PD-1 or CD107a. L/D, live/dead viability dye.

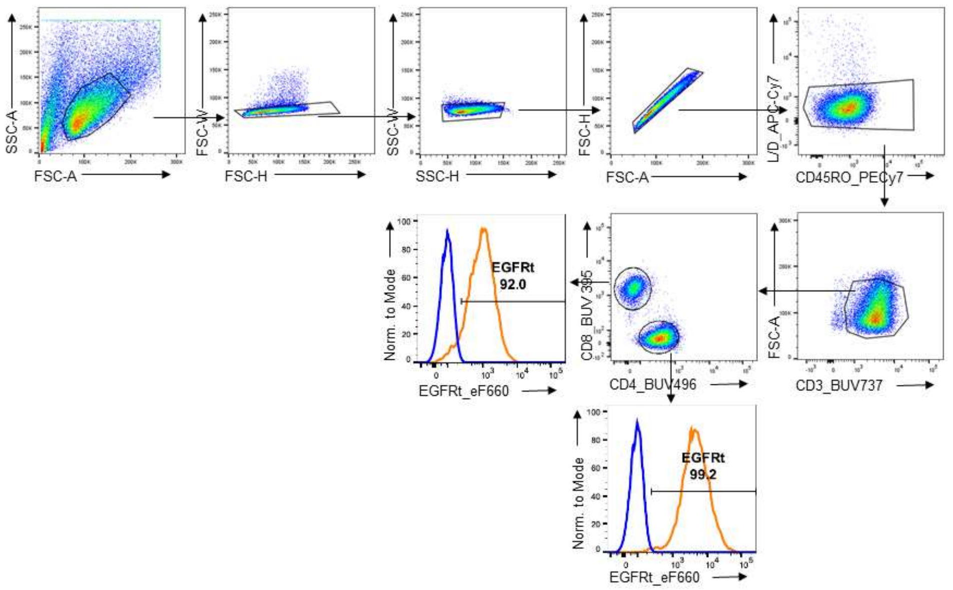

Supplementary Figure 2: Flow cytometry gating strategy for CAR expression. After setting pre-gates depicted in Supplementary Figure 1, $CD4^+$ and $CD8^+$ T cells were gated for EGFR. Gating strategy was used for Figure 1b and 2a.

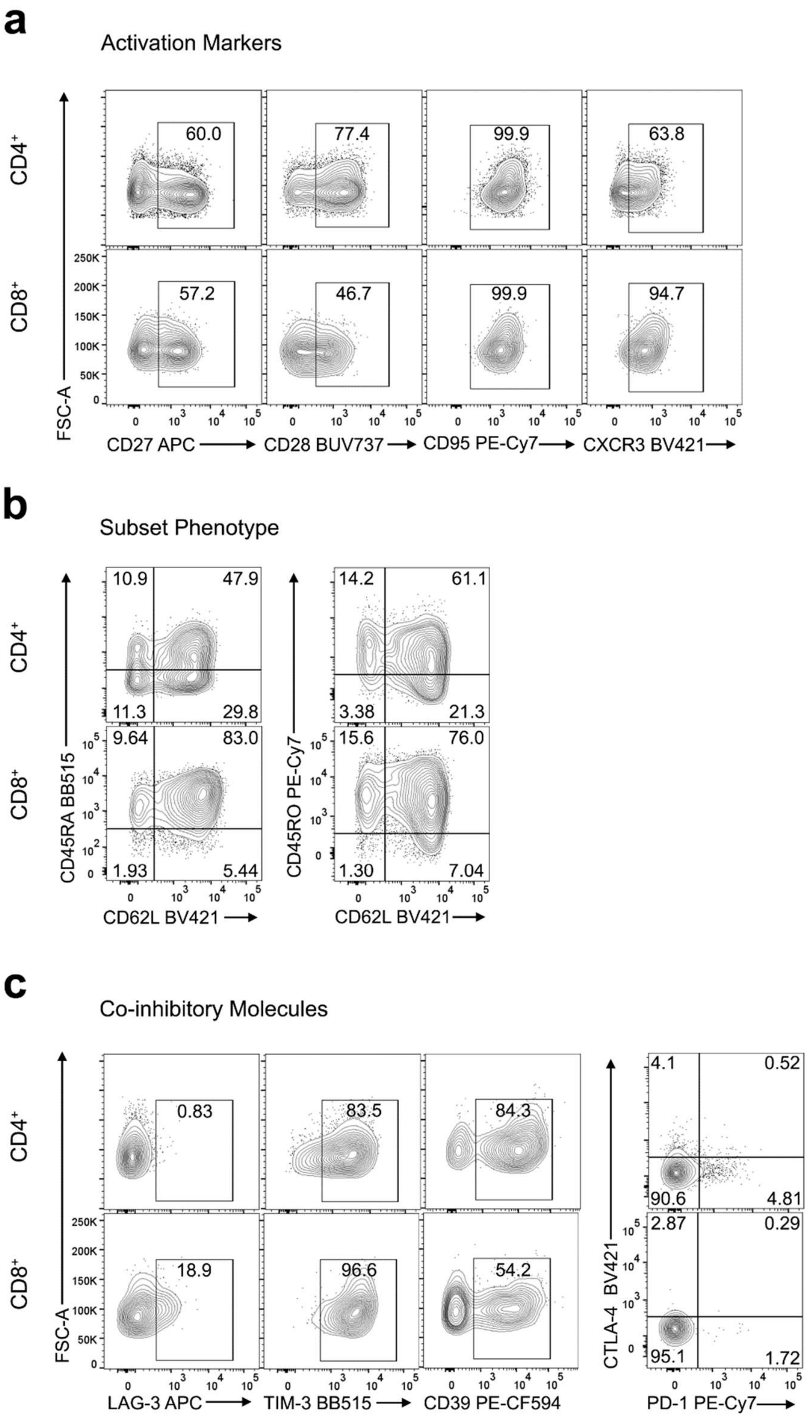

Supplementary Figure 3: Flow cytometry gating strategy for T cell phenotype. After setting pre-gates depicted in Supplementary Figure 1, CD4$^+$ and CD8$^+$ T cells were gated for **(a)** activation marker expression, **(b)** subset phenotype and **(c)** co-inhibitory molecule expression. Gates were set on fluorescence minus one (FMO) controls, with the exception of CTLA-4, which was gated based on an FMO for cultured T cells or an isotype control for tumour suspensions. Gating strategy was used for Figures 6c-d and 7c-d.

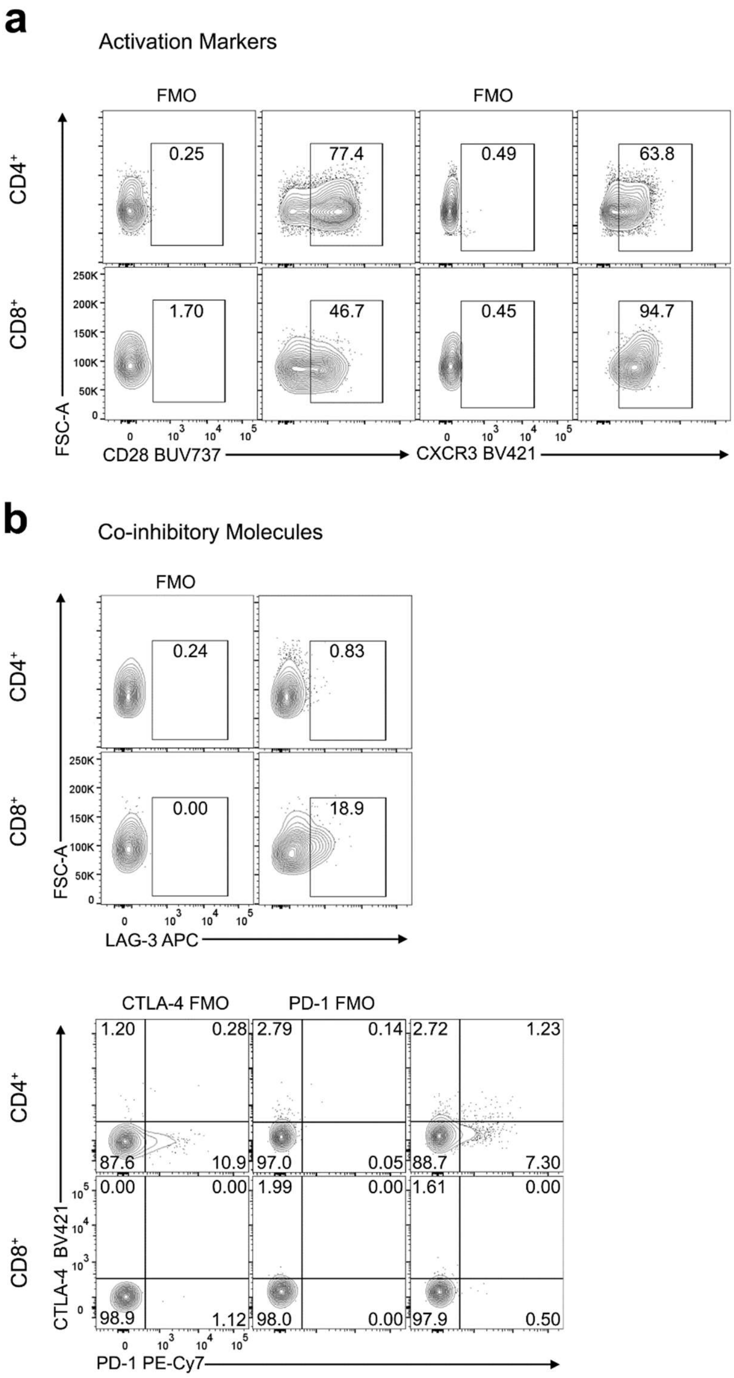

Supplementary Figure 4: Flow cytometry gating strategy for T cell phenotype (FMOs). After setting pre-gates depicted in Supplementary Figure 1, $CD4^+$ and $CD8^+$ T cells were gated for **(a)** activation marker expression and **(b)** co-inhibitory molecule expression. FMO controls are shown alongside stained samples. Gating strategy was used for Figures 4c-d and 5c-d. FMO, fluorescence minus one.

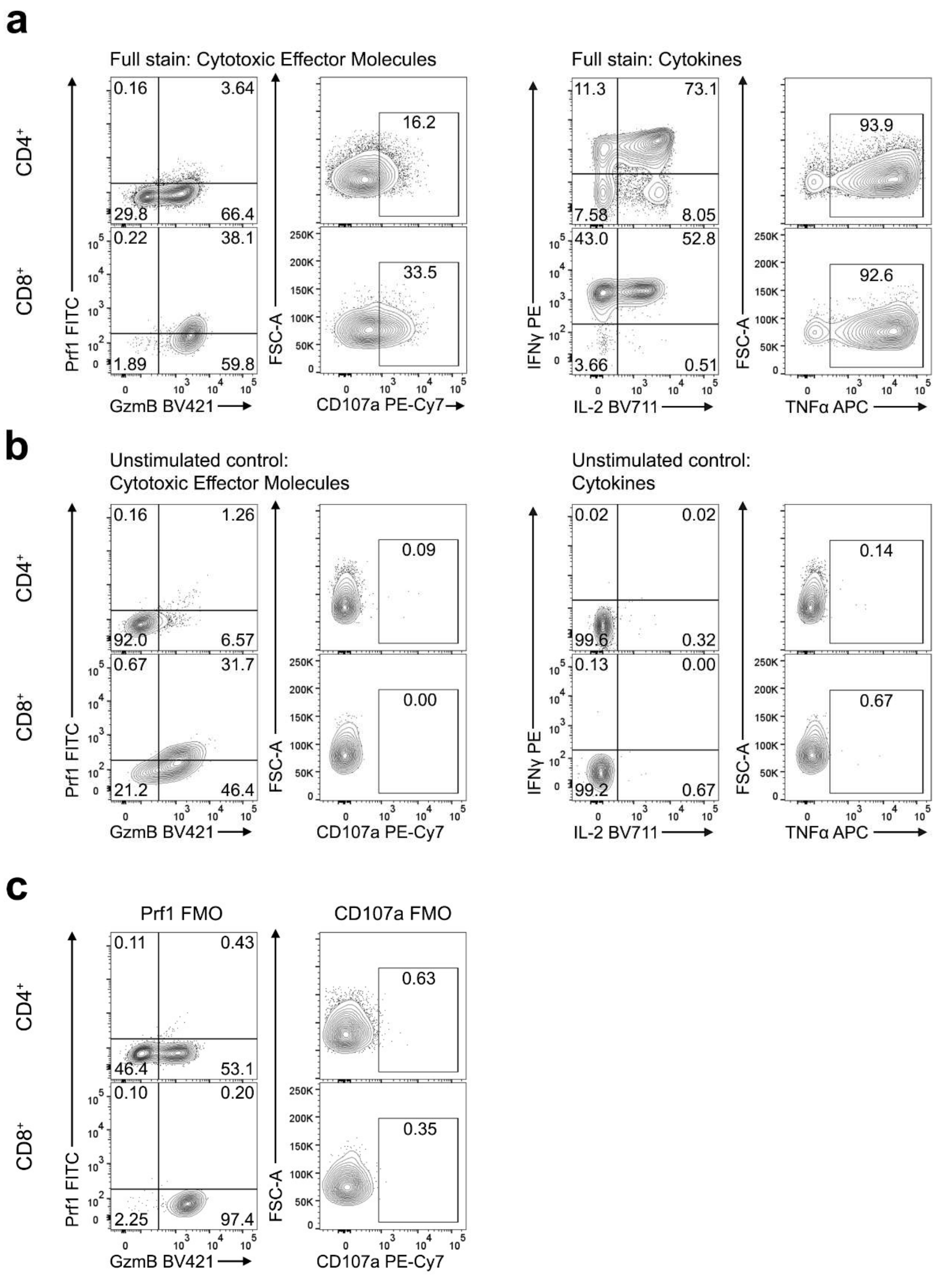

Supplementary Figure 5: Flow cytometry gating strategy for cytotoxic molecule and cytokine expression. After setting pre-gates depicted in Supplementary Figure 1, **(a)** $CD4^+$ and $CD8^+$ T cells were gated for cytotoxic molecule and cytokine expression. Gates were set on a **(b)** unstimulated control and **(c)** FMO controls. GzmB, granzyme B; Prf1, perforin; FMO, fluorescence minus one.

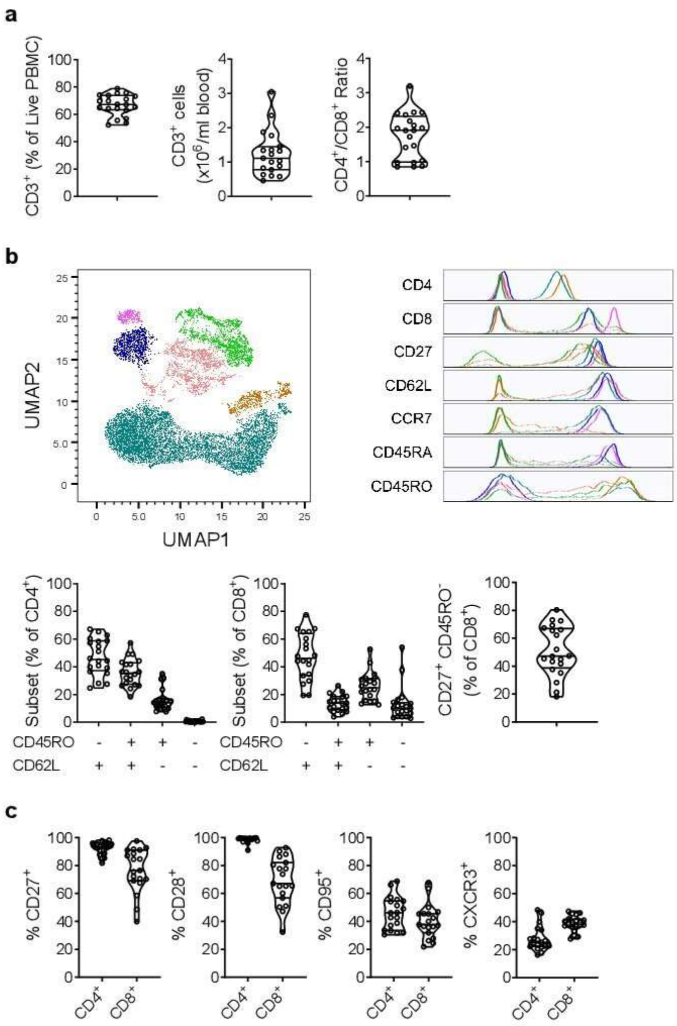

Supplementary Figure 6: Phenotyping of peripheral blood T cells isolated from healthy donors.

PBMCs were isolated from the fresh peripheral blood of healthy individuals and were assessed for differentiation and activation marker expression by flow cytometry. **(a)** Frequency of $CD3^+$ cells of total live PBMC (n = 20), number of $CD3^+$ cells per ml of blood (n = 19) and $CD4^+/CD8^+$ ratio of $CD3^+$ (n = 20). **(b)** UMAP analysis of differentiation/activation marker expression by $CD3^+$ cells, represented as two-dimensional clusters and histogram overlays of individual clusters; n = 20. Frequencies of T cell subset phenotypes of $CD4^+$ and $CD8^+$ and frequency of $CD27^+$ $CD45RO^-$ of $CD8^+$; n = 20. **(c)** Frequencies of activation markers, CD27, CD28, CD95 and CXCR3 of $CD4^+$ and $CD8^+$; n = 19. Gating strategy is shown in Supplementary Figures 1-3. Data pooled from 20 donors across three independent experiments and represented as mean ± SEM. PBMC, peripheral blood mononuclear cells; UMAP, uniform manifold approximation and projection.

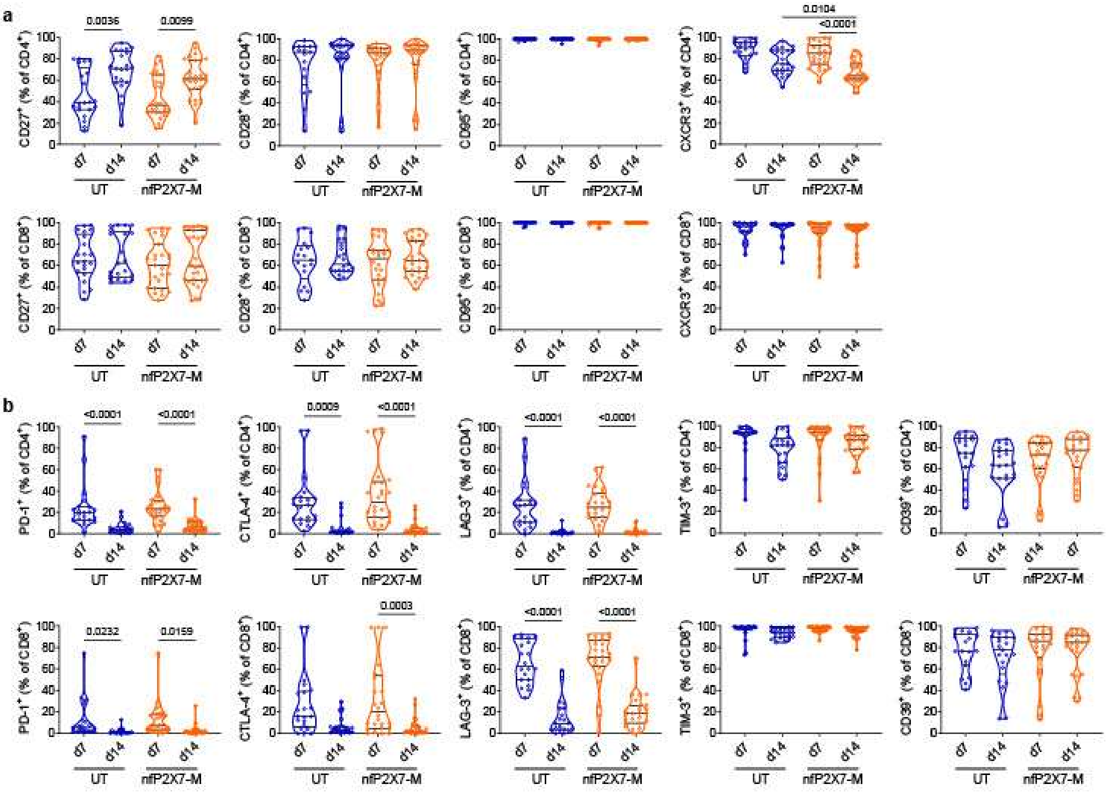

Supplementary Figure 7: NfP2X7-targeting CAR-T cells are highly activated and display minimal co-inhibitory molecule expression. NfP2X7-targeting CAR-T cells were generated and stimulated with α-CD3, α-CD28 and irradiated PBMC. **(a)** Frequency of CD27, CD28, CD95 and CXCR3 of $CD4^+$ and $CD8^+$ on d7 post-PBMC and d14 post-PBMC; n = at least 17 (UT d7), n = 21 (UT d14), n = at least 24 (nfP2X7-M d7), n = 27 (nfP2X7-M d14). **(b)** Frequency of PD-1, CTLA-4, LAG-3, TIM-3 and CD39 of $CD4^+$ and $CD8^+$ on d7 post-PBMC and d14 post-PBMC; n = at least 19 (UT d7), n = at least 20 (UT d14), n = at least 23 (nfP2X7-M d7), n = at least 25 (nfP2X7-M d14). Gating strategy is shown in Supplementary Figures 1-3. Data pooled from 10 independent CAR-T cell preparations, derived from 8 healthy donors. Data represented as mean ± SEM; Ordinary one-way ANOVA with Bonferroni's post-test.

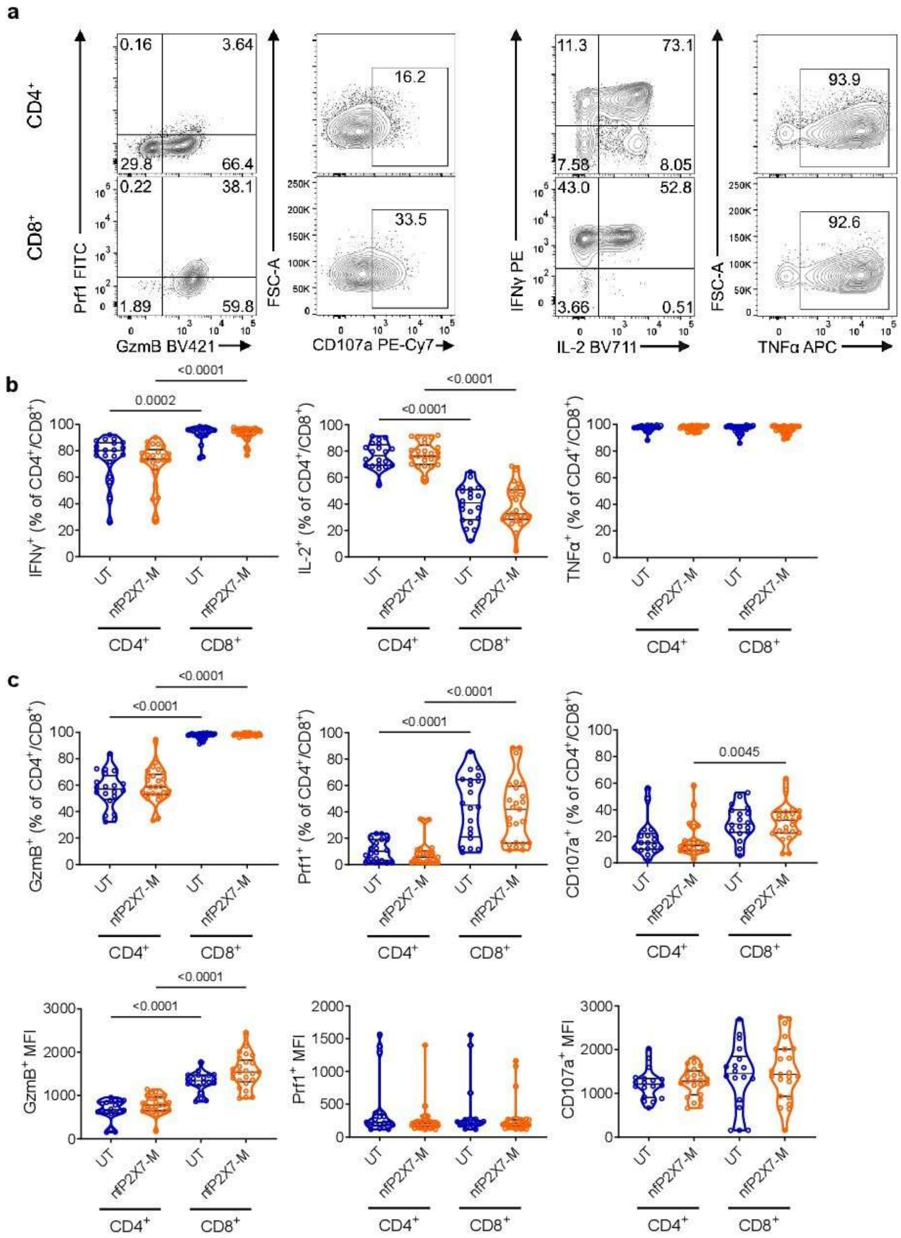

Supplementary Figure 8: NfP2X7-targeting CAR-T cells express cytotoxic molecules and T_H1 cytokines. NfP2X7-targeting CAR-T cells were generated and stimulated with α-CD3, α-CD28 and irradiated PBMC. On d14 post-PBMC, cells were assessed by flow cytometry following PMA stimulation. **(a)** Representative flow cytometry on d14 post-PBMC. **(b)** Frequency of cytokine expression of $CD4^+$ and $CD8^+$; n = 20 (UT), n = 25 (nfP2X7-M). **(c)** Frequency and geometric fluorescence intensity (gMFI) of granzyme B, perforin and CD107a expression of $CD4^+$ and $CD8^+$; n = at least 18 (UT), n = at least 23 (nfP2X7-M). Gating strategy is shown in Supplementary Figures 1 and 4. Data pooled from 10 independent CAR-T cell preparations, derived from 8 healthy donors. Data represented as mean ± SEM; Ordinary one-way ANOVA with Bonferroni's post-test. GzmB, granzyme B; Prf1, perforin; PMA, phorbol 12-myristate 13-acetate.

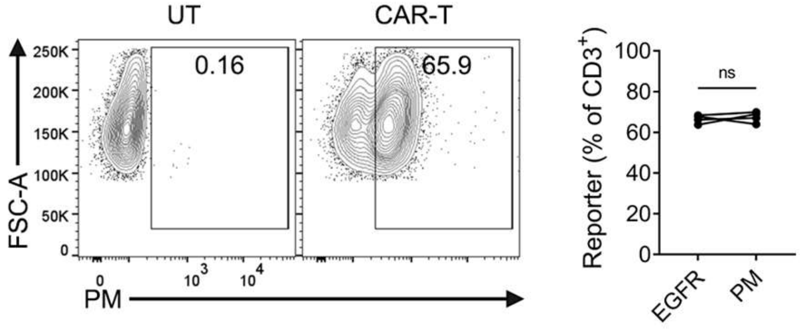

Supplementary Figure 9: EGFRt reporter expression is a reliable measure of CAR expression. Frozen nfP2X7-M CAR-T cells were thawed and rested in IL-2 and IL-15 for 2-3 days. (a) Representative flow cytometry and (b) frequency of CAR^+ cells, based on EGFR expression and nfP2X7 peptide mimetic staining of the same cell population, pre-gated on $CD3^+$; n = 4; two-tailed paired *t*-test. Data in (b) pooled from four independent experiments. PM, peptide mimetic; SA, streptavidin; ns, non-significant.

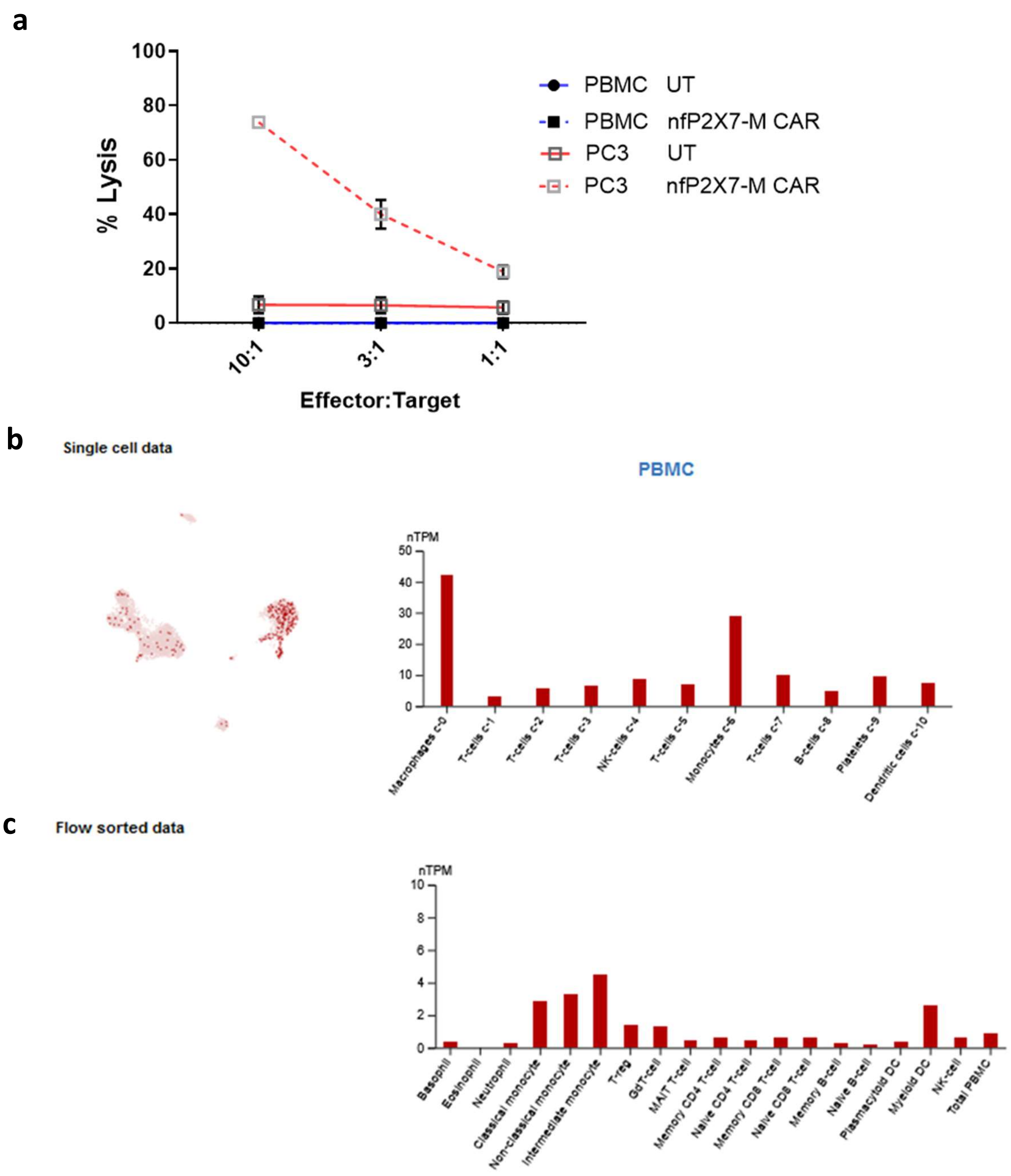

Supplementary Figure 10: NfP2X7-targeting CAR-T cells do not exhibit cytotoxicity against WT P2X7 expressed by PBMC populations. (a) To demonstrate that nfP2X7-M CAR-T cells were not cytotoxic towards WT P2X7-positive healthy donor-derived normal cells, nfP2X7-M CAR-T cells or untransduced (UT) donor-matched control cells were co-cultured with healthy donor-derived PBMCs at effector:target ratios of 10:1, 3:1 and 1:1 for 18 h. As a positive control, nfP2X7-M CAR-T cells and donor-matched UT control cells were co-cultured with PC3 prostate cancer cells. Specific cytotoxicity was measured using a BrightGlo luciferase-based cytotoxicity assay system. Data from an experiment testing three independent CAR-T cell preparations prepared from three donors. Gene expression datasets sourced from the Human Protein Atlas database (https://theproteinatlas.com) were analysed for expression of P2X7 RNA on **(b)** single cells from a PBMC scRNAseq dataset, with cluster map and **(c)** different FACS-sorted cell populations of PBMCs.

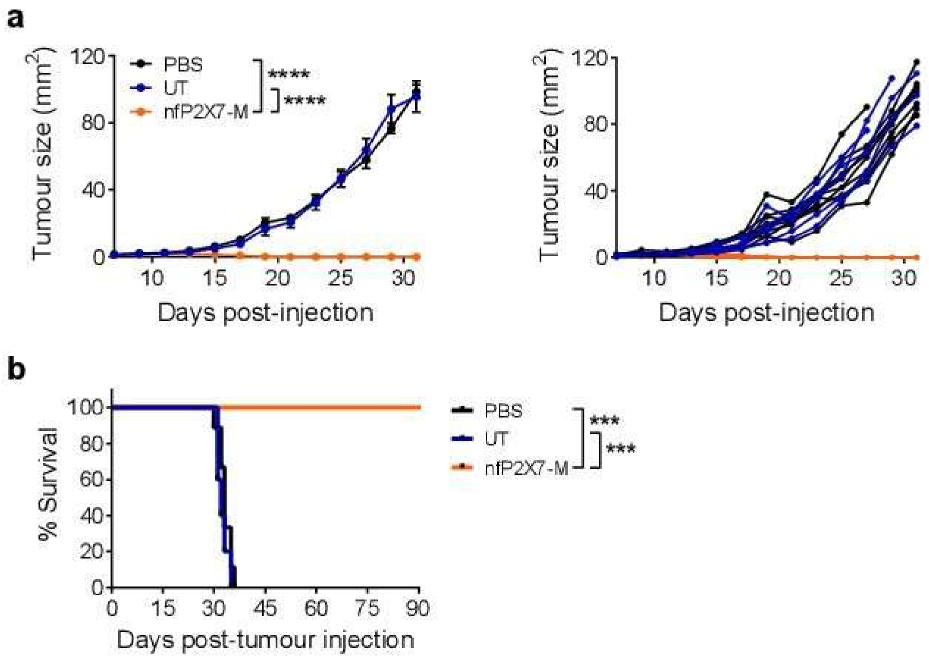

Supplementary Figure11: 6-7 week old male NOD-*scid* IL2Rγ^{null} (NSG) mice were subcutaneously injected with 1x10^6 PC3 human prostate cancer cells into the lower abdomen and intravenously injected with 2x10^7 nfP2X7-targeting CAR-T cells or untransduced T cells on d3 post-tumour injection. **(a)** Tumour growth curves as pooled and individual mice; two-way ANOVA with Bonferroni's post-test, **** $p<0.0001$. **(b)** Kaplan-Meier survival curve; Mantel-Cox test, *** $p=0.0009$. Data from a single experiment and represented as mean ± SEM; n = 5 (UT), n = 5 (nfP2X7-M).

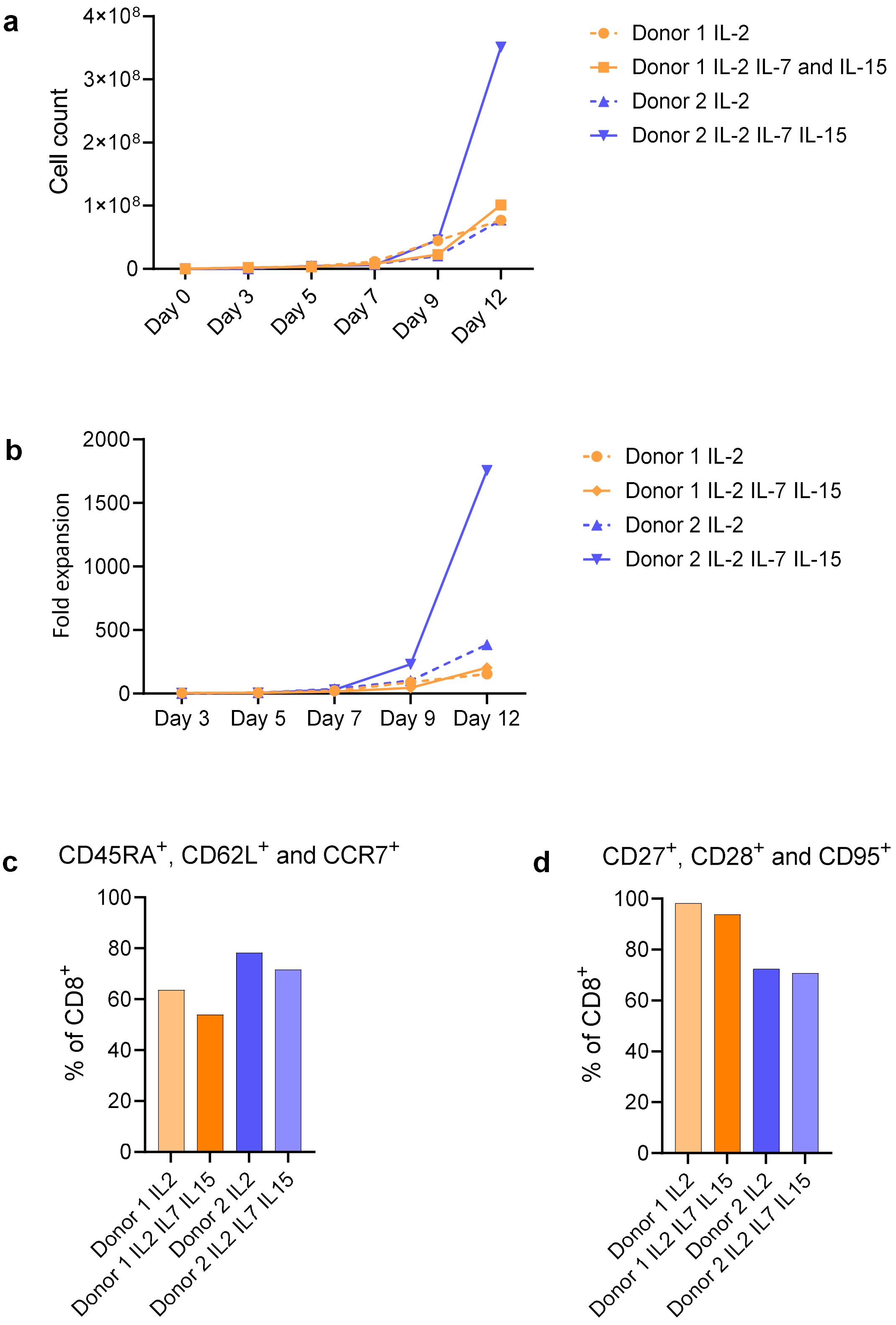
a
Cell count
4×10^8
3×10^8
2×10^8
1×10^8
0
Day 0
Day 3
Day 5
Day 7
Day 9
Day 12
Donor 1 IL-2
Donor 1 IL-2 IL-7 and IL-15
Donor 2 IL-2
Donor 2 IL-2 IL-7 IL-15
b
Fold expansion
2000
1500
1000
500
0
Day 3
Day 5
Day 7
Day 9
Day 12
Donor 1 IL-2
Donor 1 IL-2 IL-7 IL-15
Donor 2 IL-2
Donor 2 IL-2 IL-7 IL-15
c
CD45RA+, CD62L+ and CCR7+
% of CD8+
100
80
60
40
20
0
Donor 1 IL2
Donor 1 IL2 IL7 IL15
Donor 2 IL2
Donor 2 IL2 IL7 IL15
d
CD27+, CD28+ and CD95+
% of CD8+
100
80
60
40
20
0
Donor 1 IL2
Donor 1 IL2 IL7 IL15
Donor 2 IL2
Donor 2 IL2 IL7 IL15

Supplementary Figure 12: Enhanced CAR-T cell growth using IL-2, IL-7 and IL-15. NfP2X7 targeting CAR-T cells generated from two donors were cultured in media supplemented with IL-2 only or a combination of IL-2, IL-7 and IL-15. **a)** Comparison of nfP2X7 targeting CAR-T cell numbers over a 12 day expansion, cultured in IL-2 only or a combination of IL-2, IL-7 and IL-15 **b)** Comparison of nfP2X7 targeting CAR-T cell fold expansion over a 12 day expansion, cultured in IL-2 only or a combination of IL-2, IL-7 and IL-15. **c)** Percentage of cells positive for $CD45RA^+$, $CD62L^+$ and $CCR7_+$ at the end of the 12 day expansion, cultured in IL-2 only or a combination of IL-2, IL-7 and IL-15. **d)** Percentage of cells positive for $CD27^+$, $CD28^+$ and $CD95^+$ at the end of the 12 day expansion, cultured in IL-2 only or a combination of IL-2, IL-7 and IL-15.

Supplementary Table 1 Details of cancer cell lines used in this study

Cell line name	Cancer type	Media	Obtained from
PC9	Adenocarcinoma	RPMI 1640 media (Sigma Aldrich) supplemented with 2mM L-glutamine (Gibco) and 10% foetal bovine serum (Scientifix)	CellBank Australia, the European Collection of Cell Cultures
K562,	Leukemia	RPMI 1640 media (Sigma Aldrich) supplemented with 10% foetal bovine serum (Scientifix)	American Type Culture Collection
K562-OKT3	Leukemia	RPMI 1640 media (Sigma Aldrich) supplemented with 10% foetal bovine serum (Scientifix)	Kindly provided by Prof Michale Jensen (Seattle Children's Research Institute, Seattle).
K562-Luc	Leukemia	RPMI 1640 media (Sigma Aldrich) supplemented with 10% foetal bovine serum (Scientifix)	CellBank Australia, Japanese Collection of Research Bioresources Cell Bank
MDA-MB-231	Breast	DMEM media (Sigma Aldrich) supplemented with 10% foetal bovine serum (Scientifix)	The human triple negative breast adenocarcinoma MDA-MB-231 cell line used in *in vivo* experiments was kindly provided by Prof Robin Anderson (Olivia Newton-John Cancer Research Institute, VIC).
MDA-MB-231-Luc	Breast	DMEM media (Sigma Aldrich) supplemented with 10% foetal bovine serum (Scientifix)	CellBank Australia, Japanese Collection of Research Bioresources Cell Bank
MDA-MB-231-LM2	Breast	DMEM media (Sigma Aldrich) supplemented with 10% foetal bovine serum (Scientifix)	Gifted from J Massague, Sloan Kettering Institute
BT-549-Luc	Breast	RPMI 1640 media (Sigma Aldrich) supplemented with 10% foetal bovine serum (Scientifix) and 0.023IU Insulin (Protaphone)	CellBank Australia, Japanese Collection of Research Bioresources Cell Bank
HCT-116	Colorectal	DMEM media (Sigma Aldrich) supplemented with 2 mM L-glutamine (Gibco) and 10% foetal bovine serum (Scientifix)	CellBank Australia, the European Collection of Cell Cultures
Lim-1215	Colorectal	RPMI 1640 media (Sigma Aldrich) supplemented with 2mM L-glutamine (Gibco), 10% foetal bovine serum (Scientifix), 25mM Hepes, 0.6mg/mL Insulin	CellBank Australia

		(Protaphone), 1mg/mL Hydrocortisone (Sigma) and 10mM 1-Thioglycerol (Merck)	
LOVO	Colorectal	Ham's F12 media (Sigma Aldrich) supplemented with 10% foetal bovine serum (Scientifix)	European Collection of Cell Cultures, purchased from CellBank Australia
U87	Glioma	EMEM (EBSS) + 2mM Glutamine + 1% Non-Essential Amino Acids (NEAA) + 1mM Sodium Pyruvate (NaP) + 10% Foetal Bovine Serum (FBS).	American Type Culture Collection
UM-SCC-1	Head and neck squamous carcinoma	RPMI 1640 media (Sigma Aldrich) supplemented with 10% foetal bovine serum (Scientifix)	American Type Culture Collection
NCI-H460-Luc	Lung	RPMI 1640 media (Sigma Aldrich) supplemented with 10% foetal bovine serum (Scientifix)	CellBank Australia, Japanese Collection of Research Bioresources Cell Bank
Namalwa	Lymphoma	RPMI 1640 media (Sigma Aldrich) supplemented with 2mM L-glutamine (Gibco) and 10% foetal bovine serum (Scientifix)	CellBank Australia, the European Collection of Cell Cultures
Raji	Lymphoma	RPMI 1640 media (Sigma Aldrich) supplemented with 2mM L-glutamine (Gibco) and 10% foetal bovine serum (Scientifix)	CellBank Australia, the European Collection of Cell Cultures
Ramos	Lymphoma	RPMI 1640 media (Sigma Aldrich) supplemented with 2mM L-glutamine (Gibco) and 10% foetal bovine serum (Scientifix)	CellBank Australia, the European Collection of Cell Cultures
C32	Melanoma	RPMI 1640 media (Sigma Aldrich) supplemented with 10% foetal bovine serum (Scientifix)	American Type Culture Collection
M21	Melanoma		American Type Culture Collection
Skmel-5	Melanoma	RPMI 1640 media (Sigma Aldrich) supplemented with 10% foetal bovine serum (Scientifix)	American Type Culture Collection
Skmel-28	Melanoma	RPMI 1640 media (Sigma Aldrich) supplemented with 10% foetal bovine serum (Scientifix)	American Type Culture Collection

Be(2)-M17	Neuroblastoma	Ham's F12/ EMEM media (both Sigma Aldrich) supplemented with 1% NEAA (Life Technologies), 2 mM L-glutamine (Gibco) and 15% foetal bovine serum (Scientifix)	CellBank Australia, the European Collection of Cell Cultures
Kelly	Neuroblastoma	RPMI 1640 media (Sigma Aldrich) supplemented with 2mM L-glutamine (Gibco) and 10% foetal bovine serum (Scientifix)	CellBank Australia, the European Collection of Cell Cultures
SH-SY-5Y	Neuroblastoma	Ham's F12 / EMEM (ATCC) media supplemented with 1% NEAA (Life Technologies), 2 mM L-glutamine (Gibco) and 15% foetal bovine serum (Scientifix)	CellBank Australia, the European Collection of Cell Cultures
SK-N-DZ	Neuroblastoma	DMEM media (Sigma Aldrich) supplemented with 2 mM L-glutamine (Gibco), 20% foetal bovine serum (Scientifix) and 1% NEAA (Sigma Aldrich)	CellBank Australia, the European Collection of Cell Cultures
OVCAR3-Luc	Ovarian	RPMI 1640 media (Sigma Aldrich) supplemented with 15U Insulin (Protaphone), 20% foetal bovine serum (Scientifix)	CellBank Australia, Japanese Collection of Research Bioresources Cell Bank
OVCAR5	Ovarian	RPMI 1640 media (Sigma Aldrich) supplemented with 2 mM L-glutamine (Gibco) and 10% foetal bovine serum (Scientifix)	Dr Thomas Hamilton (Fox Chase Cancer Center, PA, USA)
AsPC-1/CMV-Luc	Pancreatic	RPMI 1640 media (Sigma Aldrich) supplemented with 10% foetal bovine serum (Scientifix)	CellBank Australia, Japanese Collection of Research Bioresources Cell Bank
PC3-Luc	Prostate	Ham's F12 media supplemented with 7% foetal bovine serum (Scientifix)	CellBank Australia, the European Collection of Cell Cultures
DU-145-luc	Prostate	EMEM media (Sigma Aldrich) supplemented with 10% foetal bovine serum (Scientifix)	CellBank Australia, Japanese Collection of Research Bioresources Cell Bank
RPMI-8226	Myeloma	RPMI 1640 media (Sigma Aldrich) supplemented with 10% foetal bovine serum (Scientifix)	Kindly provided by Dr Krzysztof Mrozik, University of Adelaide
RD	Rhabdomyosarcoma	DMEM media (Sigma Aldrich) supplemented with 2 mM L-glutamine (Gibco), 10% foetal bovine serum (Scientifix), 2%	CellBank Australia, the European Collection of Cell Cultures

		NEAA (Life Technologies) and 2% Vitamins (Gibco)	

Supplementary Table 2 Monoclonal antibodies and related reagents

**Primary antibodies for flow cytometry and cell culture**					
Antigen	Conjugate	Clone	Manufacturer/Cat .No.	Final concentration or dilution from manufacturer's test size	Final dilution
CD3	BUV737<br>Purified (cell culture)	UCHT1<br>OKT3	BD<br>/612750	30ng/ml<br>1/8	1:250
CD4	BUV496	SK3	BD/612936	1/4	1:200
CD8	BUV395	RPA-T8	BD/563795	1/8	1:250
Granzyme B	BV421	GB11	BD/563389	1/2	1:100
Perforin	FITC	B-D48	Biolegend/353310	1/4	1:200
CD107a	PECy7	H4A3	BD/561348	1/6	1:300
IFNγ	PE	B27	BD/559327	Recommended	1:12.5
TNFα	APC	MAb11	BD/554514	1/2	1:200
IL-2	BV711	5344.111	BD/563946	1/4	1:50
CCR7	PE	150503<br>2-L1-A	BD/560765<br>BD/566741	Recommended<br>Recommended	1:12.5
CD45RA	BB515	HI100	BD/564552	1/6	1:250
CD45RO	PECy7	UCHL1	BD/337168	Recommended	1:50
CD62L	BV421	DREG-56	BD/563862	1/2	1:15
EGFR	eF660	me1B3	Invitrogen/50-9509-42	Recommended	1:50

CD27	APC	M-T271	BD/558664	Recommend ed	1:25
CD28	BUV737	CD28.2	BD/612815	Recommend ed	1:50
CD95	PECy7	DX2	BD/561633	1/8	1:200
CXCR3	BV421	1C6/CXCR 3	BD/562558	Recommend ed	1:50
PD-1	PECy7	EH12.1	BD/561272	Recommend ed	1:50
CTLA-4	BV421	BNI3	BD/562743	Recommend ed	1:50
TIM-3	BB515	7D3	BD/565588	Recommend ed	1:50
LAG-3	AF-647	T47-530	BD/565716	Recommend ed	1:50
CD39	PE-CF594<br>BUV805	TU66	BD/563678	Recommend ed	1:50
IgG2a isotype	BV421	G155-178	BD	1/10	
**Related flow cytometry reagents**					
Reagent	Conjugate	Clone	Manufacturer	Final dilution	
Fixable Viability Dye	780	-	BD/565388	1/1000	1:1000
NfP2X7 Peptide Mimetic	Biotin	-	GenScript	400nM	
Streptavidin	BV421	0.5µg/ml	BD	1:200	
