# Supplementary material for: Pre-clinical validation of a pan-cancer CAR-T cell immunotherapy targeting nfP2X7
Source: Nat Commun. 2023 Sep 8;14:5546. doi: 10.1038/s41467-023-41338-y (PMC10491676; doi:10.1038/s41467-023-41338-y)
Supplement: Supplementary file 2 — Description of Additional Supplementary Files [file 41467_2023_41338_MOESM2_ESM.docx]

**Description of Additional Supplementary Files**

File name: **Supplementary Movie 1**

Description: **Real-time imaging of nfP2X7-M CAR-T cell adhesion to a blood vessel *in vivo*.** For intravital microscopy, 2x10^6^ MDA-MB-231-LM2 cells expressing GFP were injected into the mammary fat pad of NSG mice and allowed to establish for 2 weeks. The mice then received an intravenous injection of 2x10^7^ nfP2X7-M CAR-T cells fluorescently labelled with CellTracker Orange as well as Cy5 Dextran (for visualisation of blood vessels with active blood flow). The surgically exposed tumour was then placed under a Zeiss LSM710-NLO microscope with a 20x objective lens and the interaction between the CAR T cells and the blood vessels were visualised in real time. Representative of two independent experiments. Scale bars = 50 µm.

File name: **Supplementary Movie 2**

Description: **Real-time imaging of nfP2X7-M CAR-T cell and transmigration through blood vessels *in vivo*.** For intravital microscopy, 2x10^6^ MDA-MB-231-LM2 cells expressing GFP were injected into the mammary fat pad of NSG mice and allowed to establish for 2 weeks. The mice then received an intravenous injection of 2x10^7^ nfP2X7-M CAR-T cells fluorescently labelled with CellTracker Orange as well as Cy5 Dextran (for visualisation of blood vessels with active blood flow). The surgically exposed tumour was then placed under a Zeiss LSM710-NLO microscope with a 20x objective lens and the interaction between the CAR T cells and the blood vessels were visualised in real time. Representative of two independent experiments. Scale bars = 50 µm.

File name: **Supplementary Movie 3**

Description: **Real-time imaging of nfP2X7-M CAR-T cells killing MDA-MB-231 breast cancer cells *in vitro*.** MDA-MB-231 breast cancer cells were seeded into wells and allowed to grow overnight. nfP2X7-M CAR-T cells pulsed with Fluo-4 AM (green), and PI (red) were added into the wells at the same time and are seen to directly engage with MDA-MB-231 cells and promote cell death. Images were taken at 10s intervals for at least 3 hours using a 40x objective lens on a Zeiss LSM700 and compiled for time lapse video. Representative of three independent experiments.

File name: **Supplementary Movie 4**

Description: **Real-time imaging of untransduced cells interaction with MDA-MB-231 breast cancer cells *in vitro*.** MDA-MB-231 breast cancer cells were seeded into wells and allowed to grow overnight. Untransduced (UT) T cells pulsed with Fluo-4 AM (green), and PI (red) were added into the wells at the same time and exhibited almost no tumour cell engagement and cytotoxicity. Images were taken at 10s intervals for at least 3 hours using a 40x objective lens on a Zeiss LSM700 and compiled for time lapse video. Representative of three independent experiments.
