# Supplementary material for: Pre-clinical validation of a pan-cancer CAR-T cell immunotherapy targeting nfP2X7
Source: Nat Commun. 2023 Sep 8;14:5546. doi: 10.1038/s41467-023-41338-y (PMC10491676; doi:10.1038/s41467-023-41338-y)
Supplement: Supplementary file 7 — Reporting Summary [file 41467_2023_41338_MOESM7_ESM.pdf]

nature portfolio

Corresponding author(s): Simon C Barry

Last updated by author(s): 19/07/2023

# Reporting Summary

Nature Portfolio wishes to improve the reproducibility of the work that we publish. This form provides structure for consistency and transparency in reporting. For further information on Nature Portfolio policies, see our Editorial Policies and the Editorial Policy Checklist.

## Statistics

For all statistical analyses, confirm that the following items are present in the figure legend, table legend, main text, or Methods section.

| n/a | Confirmed | |
|---|---|---|
| ☐ | ☒ | The exact sample size (*n*) for each experimental group/condition, given as a discrete number and unit of measurement |
| ☐ | ☒ | A statement on whether measurements were taken from distinct samples or whether the same sample was measured repeatedly |
| ☐ | ☒ | The statistical test(s) used AND whether they are one- or two-sided<br>*Only common tests should be described solely by name; describe more complex techniques in the Methods section.* |
| ☒ | ☐ | A description of all covariates tested |
| ☐ | ☒ | A description of any assumptions or corrections, such as tests of normality and adjustment for multiple comparisons |
| ☐ | ☒ | A full description of the statistical parameters including central tendency (e.g. means) or other basic estimates (e.g. regression coefficient) AND variation (e.g. standard deviation) or associated estimates of uncertainty (e.g. confidence intervals) |
| ☒ | ☐ | For null hypothesis testing, the test statistic (e.g. *F*, *t*, *r*) with confidence intervals, effect sizes, degrees of freedom and *P* value noted<br>*Give P values as exact values whenever suitable.* |
| ☒ | ☐ | For Bayesian analysis, information on the choice of priors and Markov chain Monte Carlo settings |
| ☒ | ☐ | For hierarchical and complex designs, identification of the appropriate level for tests and full reporting of outcomes |
| ☒ | ☐ | Estimates of effect sizes (e.g. Cohen's *d*, Pearson's *r*), indicating how they were calculated |

*Our web collection on statistics for biologists contains articles on many of the points above.*

## Software and code

Policy information about availability of computer code

| | |
|---|---|
| Data collection | Chromium release based cytotoxicity assay raw data acquisition was performed on the Perkin Elmer TopCount scintillation counter. Promega GloMax software was used for luminescence based cytotoxicity raw data acquisition. Realtime impedance based cytotoxicity assay data was acquired using the xCELLigence® RTCA DP instrument software (ACEA Biosciences). Beckton Dickinson Facsdiva software was used for Flow cytometry raw data acquisition. Data acquisition from the LSM 700 laser scanning confocal microscope (Zeiss) and the LSM710 Two Photon Confocal Laser Scanning Microscope (Zeiss) used the Zen 2011 (black edition) software (Zeiss). |
| Data analysis | Flow cytometry data analysis was performed using FlowJo Software V.10 (TreeStar, BD Biosciences). Graphpad Prism was used for all other data analysis and visualisation. |

For manuscripts utilizing custom algorithms or software that are central to the research but not yet described in published literature, software must be made available to editors and reviewers. We strongly encourage code deposition in a community repository (e.g. GitHub). See the Nature Portfolio guidelines for submitting code & software for further information.

## Data

Policy information about availability of data

All manuscripts must include a data availability statement. This statement should provide the following information, where applicable:

- Accession codes, unique identifiers, or web links for publicly available datasets
- A description of any restrictions on data availability
- For clinical datasets or third party data, please ensure that the statement adheres to our policy

All data supporting the findings in this study are provided in the source data file. Data availability statement included in manuscript.

## Research involving human participants, their data, or biological material

Policy information about studies with human participants or human data. See also policy information about sex, gender (identity/presentation), and sexual orientation and race, ethnicity and racism.

| | |
|---|---|
| Reporting on sex and gender | As anonymous random donors were used for PBMC in this study, we did not collect sex and gender information |
| Reporting on race, ethnicity, or other socially relevant groupings | As anonymous random donors were used for PBMC in this study, we did not collect race, ethnicity or other socially relevant groupings information. |
| Population characteristics | As anonymous  healthy random donors were recruited, we did not collect population characteristics. |
| Recruitment | Anonymous  healthy random donors were recruited |
| Ethics oversight | Human CD3+ T cells were isolated from whole blood or buffy coats obtained from anonymous healthy donors under consent Human CD3 T cells were collected as approved by HREC and governance committees at WCHN Adelaide(SSA/19/WCHN/96) (HEC/19/WCHN/65). |

Note that full information on the approval of the study protocol must also be provided in the manuscript.

# Field-specific reporting

Please select the one below that is the best fit for your research. If you are not sure, read the appropriate sections before making your selection.

☒ Life sciences ☐ Behavioural & social sciences ☐ Ecological, evolutionary & environmental sciences

For a reference copy of the document with all sections, see nature.com/documents/nr-reporting-summary-flat.pdf

# Life sciences study design

All studies must disclose on these points even when the disclosure is negative.

| | |
|---|---|
| Sample size | For human donor  peripheral blood samples, no sample size calculation was required, as a representative sample size for batch variation was sufficient for proof of concept. For animal studies a sample size of 7 mice per treatment group was used, as per standard animal experimental design principles. |
| Data exclusions | none |
| Replication | Experiments were performed in triplicate, or more as stated in the methods and figure legends. |
| Randomization | Samples of PBMC were randomly allocated to control or CAR-T manufacture. |
| Blinding | Staff who performed animal studies were blinded to the treatment group during acquisition and analysis,  and this was maintained until the endpoint was reached. |

# Reporting for specific materials, systems and methods

We require information from authors about some types of materials, experimental systems and methods used in many studies. Here, indicate whether each material, system or method listed is relevant to your study. If you are not sure if a list item applies to your research, read the appropriate section before selecting a response.

## Materials & experimental systems

| n/a | Involved in the study |
|---|---|
| ☐ | ☒ Antibodies |
| ☐ | ☒ Eukaryotic cell lines |
| ☒ | ☐ Palaeontology and archaeology |
| ☐ | ☒ Animals and other organisms |
| ☒ | ☐ Clinical data |
| ☒ | ☐ Dual use research of concern |
| ☒ | ☐ Plants |

## Methods

| n/a | Involved in the study |
|---|---|
| ☒ | ☐ ChIP-seq |
| ☐ | ☒ Flow cytometry |
| ☒ | ☐ MRI-based neuroimaging |

# Antibodies

Antibodies used

Antibodies used and the catalogue numbers are listed in supplementary table 2:

Primary antibodies for flow cytometry and cell culture
Antigen Conjugate Clone Manufacturer/Cat.No.
CD3 BUV737 Purified (cell culture) UCHT1 OKT3 BD /612750
CD4 BUV496 SK3 BD/612936
CD8 BUV395 RPA-T8 BD/563795
Granzyme B BV421 GB11 BD/563389
Perforin FITC B-D48 Biolegend/353310
CD107a PECy7 H4A3 BD/561348 1
IFNγ PE B27 BD/559327
TNFα APC MAb11 BD/554514
IL-2 BV711 5344.111 BD/563946
CCR7 PE 150503 2-L1-A BD/560765 BD/566741
CD45RA BB515 HI100 BD/564552
CD45RO PECy7 UCHL1 BD/337168
CD62L BV421 DREG-56 BD/563862
EGFR eF660 me1B3 Invitrogen/50-9509-42
CD27 APC M-T271 BD/558664
CD28 BUV737 CD28.2 BD/612815
CD95 PECy7 DX2 BD/561633
CXCR3 BV421 1C6/CXCR3 BD/562558
PD-1 PECy7 EH12.1 BD/561272
CTLA-4 BV421 BNI3 BD/562743
TIM-3 BB515 7D3 BD/565588
LAG-3 AF-647 T47-530 BD/565716
CD39 PE-CF594 BUV805 TU66 BD/563678
IgG2a isotype BV421 G155-178 BD
Related flow cytometry reagents
Reagent Conjugate Clone Manufacturer
Fixable Viability Dye 780 - BD/565388

Validation

In use validation of each antibody based on recommended dilutions by each manufacturer are listed in table 2 and listed :
CD3 BUV737 Purified (cell culture) UCHT1 OKT3 BD /612750 30ng/ml 1/8 1:250
CD4 BUV496 SK3 BD/612936 1/4 1:200
CD8 BUV395 RPA-T8 BD/563795 1/8 1:250
Granzyme B BV421 GB11 BD/563389 1/2 1:100
Perforin FITC B-D48 Biolegend/353310 1/4 1:200
CD107a PECy7 H4A3 BD/561348 1/6 1:300
IFNγ PE B27 BD/559327 Recommended 1:12.5
TNFα APC MAb11 BD/554514 1/2 1:200
IL-2 BV711 5344.111 BD/563946 1/4 1:50
CCR7 PE 150503 2-L1-A BD/560765 BD/566741 Recommended 1:12.5
CD45RA BB515 HI100 BD/564552 1/6 1:250
CD45RO PECy7 UCHL1 BD/337168 Recommended 1:50
CD62L BV421 DREG-56 BD/563862 1/2 1:15
EGFR eF660 me1B3 Invitrogen/50-9509-42 Recommended 1:50
CD27 APC M-T271 BD/558664 Recommended 1:25
CD28 BUV737 CD28.2 BD/612815 Recommended 1:50
CD95 PECy7 DX2 BD/561633 1/8 1:200
CXCR3 BV421 1C6/CXCR3 BD/562558 Recommended 1:50
PD-1 PECy7 EH12.1 BD/561272 Recommended 1:50
CTLA-4 BV421 BNI3 BD/562743 Recommended 1:50
TIM-3 BB515 7D3 BD/565588 Recommended 1:50
LAG-3 AF-647 T47-530 BD/565716 Recommended 1:50
CD39 PE-CF594 BUV805 TU66 BD/563678 Recommended 1:50

IgG2a isotype BV421 G155-178 BD 1/10

Related flow cytometry reagents
Reagent Conjugate Clone Manufacturer Final dilution
Fixable Viability Dye 780 - BD/565388 1/1000 1:1000

## Eukaryotic cell lines

Policy information about cell lines and Sex and Gender in Research

| | |
|---|---|
| Cell line source(s) | No Primary human Cell lines were used in this study. Human primary cells were anonymously donated so no sex/gender information was collected. All other cell lines are listed in supplementary table 1 |
| Authentication | ATCC or cell bank Australia certified at time of purchase by Short Tandem Repeat profiling:<br>ATCC: Perform STR profiling following ISO 9001 and ISO/IEC 17025 quality standards, and analyse data under Cell Authentication: Standardization of Short Tandem Repeat Profiling, ASN-0002-2021<br>CellBank Australia: STR loci are amplified using the PowerPlex® 16HS System (Promega). Data are analysed using GeneMapper™ ID software (ThermoFisher). |
| Mycoplasma contamination | all negative |
| Commonly misidentified lines (See ICLAC register) | none used |

## Animals and other research organisms

Policy information about studies involving animals; ARRIVE guidelines recommended for reporting animal research, and Sex and Gender in Research

| | |
|---|---|
| Laboratory animals | NOD-scid IL2R null (NSG) mice aged 6-8 weeks.<br>Mice housed at the University of South Australia Core Animal Facility. Housing: 12-hour light/dark cycle in pathogen-free conditions in sterile IVC cages. Diet: Meat Free Rat and Mouse Diet (Manufacturer, Specialty feed, cat# SF00-100), autoclaved and fed ad libitum.<br>Mice housed at the Commonwealth Scientific and Industrial Research Organisation (CSIRO) facility or Helen Mayo Animal House (HMAH) facility at the University of Adelaide (Adelaide, SA) in specific pathogen-free conditions with a 12 hour light/dark cycle.<br>Mice were fed with 2020x Teklad Global Soy Protein-Free Extruded Rodent Diet (Envigo) ad libitum. |
| Wild animals | No wild animals were used in this study |
| Reporting on sex | Experiments used gender and age-matched mice between 6-8 weeks of age and experimental and control groups were co-housed. For the MDA-MB 231 xenograft models, female mice were used, as they are preferable for breast cancer xenograft models. For PC3 Prostate cancer xenograft models male mice wee chosen as they are appropriate for prostate cancer models |
| Field-collected samples | No field collected samples were used in this study |
| Ethics oversight | Animal experiments were approved by the Animal Ethics Committee of the University of Adelaide (S/2018/007) and the University of South Australia (AEC# U35-18) |

Note that full information on the approval of the study protocol must also be provided in the manuscript.

## Flow Cytometry

### Plots

Confirm that:

☒ The axis labels state the marker and fluorochrome used (e.g. CD4-FITC).

☒ The axis scales are clearly visible. Include numbers along axes only for bottom left plot of group (a 'group' is an analysis of identical markers).

☒ All plots are contour plots with outliers or pseudocolor plots.

☒ A numerical value for number of cells or percentage (with statistics) is provided.

### Methodology

| | |
|---|---|
| Sample preparation | CAR-T cell Flow cytometry:<br>Four different antibody panels were used to assess CAR-T cell phenotype. These included surface markers used to define the stage of cell maturation (CD45RA, CD45RO, CD62L and CCR7), cytotoxicity markers (IFN-ɣ, TNF--α, IL-2, Granzyme B, Perforin and CD107a), activation markers (CD27, CD28, CD95, CXCR3) and co-inhibitory receptors/molecules (PD-1, CTLA-4, LAG-3, TIM-3, CD39). Antibodies are listed in Supplementary Table 2. |

Cultured T cells (1x105) or ex vivo single cell tumour suspensions (1x106) were stained in 96-well round-bottom plates (Corning), using antibodies and related reagents detailed in Supplementary Table 2. For intracellular cytokine and cytotoxic molecule staining, cells were first incubated at 37°C, 5% CO2 for 4 h in complete IMDM (Gibco) supplemented with 10% FCS (Sigma-Aldrich), 100U/ml penicillin/streptomycin (Gibco), 1x GlutaMAX (Gibco), 54pM β-mercaptoethanol (Sigma-Aldrich), 50ng/ml phorbol 12-myristate 13-acetate (Life Technologies), 1nM ionomycin (Life Technologies), 1/1500 GolgiStop (BD Biosciences) and 1/1000 GolgiPlug (BD Biosciences or Biolegend). To stain CD107a, a directly conjugated antibody was added at the beginning of the PMA stimulation. All subsequent incubations were performed at room temperature unless stated otherwise. Cells were washed in PBS, before being stained with Fixable Viability Stain 780 (BD Biosciences) diluted 1/1000 and blocked with Human FC Block (BD Biosciences) for 10 mins. Cells were then washed in FACS buffer (PBS 1% BSA, 0.04% azide) and stained for 30 mins with directly conjugated antibodies in Brilliant Stain Buffer (BD Biosciences). For intracellular staining, cells were incubated with Cytofix/Cytoperm (BD Biosciences) for 20 mins at 4°C, washed in Perm/Wash buffer (BD Biosciences) and stained with intracellular directly conjugated antibodies for 30 mins at 4°C. For the biotinylated nfP2X7 peptide mimetic, cells were stained with the peptide mimetic along with directly conjugated antibodies at room temperature, washed in FACS buffer and stained with streptavidin conjugated to BV421 (BD Biosciences) for 20 mins at 4°C. After staining, cells were washed once with FACS buffer, washed once with PBS or PBS 0.04% sodium azide, resuspended in PBS 1% paraformaldehyde and stored at 4°C in the dark. Stained cells were acquired on the BD LSRFortessa X-20 flow cytometer within 5 days. Beckton Dickinson FacsDiva software was used for Flow cytometry raw data acquisition. Data analysis was performed using FlowJo Software V.10 (TreeStar, BD Biosciences). Gating strategies are detailed in Supplementary Figures 1-4.

Tumour Infiltrate flow cytometry:
Tumours were excised, manually minced into small pieces and incubated in warm digest media for 1-1.5h at 37°C with continuous gentle agitation. Tumours were mixed every 15-20 min by resuspension. Digest media was prepared by supplementing DMEM (Gibco) with 5% heat-inactivated FCS (Sigma-Aldrich), 2.5mM CaCl2, 10mM HEPES (Gibco), 100U/ml penicillin/streptomycin (Life Technologies), 30U/ml DNase I (Sigma-Aldrich) and 1mg/ml collagenase IA (Sigma-Aldrich). Tumour homogenates were passed through a 70µm filter (BD Biosciences), washed in PBS and incubated in mouse red cell lysis buffer for 5min at 37°C. Cells were then washed in PBS and stained for analysis by flow cytometry

| | |
|---|---|
| Instrument | BD LSRFortessa X-20 flow cytometer; LSM710 Two Photon Confocal Laser Scanning Microscope (Zeiss); LSM700 Laser Scanning Microscope (Zeiss). |
| Software | Beckton Dickinson Facsdiva software was used for Flow cytometry raw data acquisition. Data acquisition from the LSM 700 laser scanning confocal microscope (Zeiss) and the LSM710 Two Photon Confocal Laser Scanning Microscope (Zeiss) used the Zen 2011 (black edition) software (Zeiss). Data analysis was performed using FlowJo Software V.10 (TreeStar, BD Biosciences) |
| Cell population abundance | Purity of CD3 CART cells and subset proportions is provided in supplementary figs 1-4 |
| Gating strategy | Gating strategies are detailed in Supplementary Figures 1-4. |

☒ Tick this box to confirm that a figure exemplifying the gating strategy is provided in the Supplementary Information.
